# The genus *Scaptodrosophila* Duda part I: the *brunnea* species group from the Oriental Region, with morphological and molecular evidence (Diptera, Drosophilidae)

**DOI:** 10.3897/zookeys.671.11275

**Published:** 2017-04-26

**Authors:** Yi-Qin Liu, Qing-Song Gao, Hong-Wei Chen

**Affiliations:** 1 Department of Entomology, South China Agricultural University, Tianhe, Guangzhou, 510642, China

**Keywords:** China, DNA barcoding, integrated taxonomy, *Scaptodrosophila brunnea* species group

## Abstract

Seven new species of the *Scaptodrosophila brunnea* species group are described from east Asia: *S. maculata* **sp. n.**, *S. melanogaster* **sp. n.**, *S. nigricostata* **sp. n.**, *S. nigripecta* **sp. n.**, *S. obscurata* **sp. n.**, *S. protenipenis* **sp. n.** and *S. rhina* **sp. n.** Three known species, *S. parabrunnea* (Tsacas & Chassagnard), *S. pressobrunnea* (Tsacas & Chassagnard) and *S. scutellimargo* (Duda) are redescribed. A key to all the examined species in the *brunnea* group is provided. Species delimitations have been improved by integrating the DNA sequences with morphological information. The intra- and interspecific pairwise p-distances (proportional distance) are summarized. Some nucleotide sites with fixed status in the alignment of the *COI* sequences (664 nucleotide sites in length) are used as “pure” molecular diagnostic characters to delineate species in the *brunnea* group.

## Introduction

To date, a total of 280 species (Bächli 2016) has been described in the genus *Scaptodrosophila* Duda, 1923 from around the world: four species from the Nearctic region, two species from the Neotropical region, ten species from the Palearctic region, 32 species from the Afrotropical region, 79 species from the Oriental region and 167 species from the Australasian region (Brake and Bächli 2008; Bächli 2016). So far, 12 species groups (Bächli 2016) have been established in *Scaptodrosophila*: the *albifrontata* group (Wheeler and Takada 1966), the *aterrima* group (Tsacas et al. 1988), the *barkeri* group (Bock and Parsons (1978), the *brunnea* group (Tsacas and Chassagnard 1976), the *brunneipennis* group (Bock and Parsons 1978), the *bryani* group (Throckmorton 1962), the *coracina* group (Mather 1955), the *inornata* group (Parsons and Bock 1978), the *latifasciaeformis* group (Burla 1954), the *rufifrons* group (Papp et al. 1999), the *saba* group (Burla 1954) and the *victoria* group (Wheeler 1949).

The *brunnea* group includes eleven known species (Bächli 2016), and was divided into two subgroups by Tsacasand and Chassagnard (1976): the *brunnea* subgroup including five species, all from Oriental region (scutellum yellow at tip): *S. brunnea* de Meijere, 1911, *S. parabrunnea* (Tsacas & Chassagnard, 1976), *S. pressobrunnea* (Tsacas & Chassagnard, 1976), *S. scutellimargo* (Duda, 1924), *S. kyushuensis* (Tsacas & Chassagnard, 1976); the *eoundo* subgroup including two species from Afrotropical region (scutellum pale at tip): *S. eoundo* (Tsacas & Chassagnard, 1976), *S. medleri* (Tsacas & Chassagnard, 1976). However, these two subgroups have not been mentioned since for the following species: *S. cultello* (Bock, 1982), *S. koraputae* (Gupta & Panigrahy, 1982), *S. paracultello* (Bock, 1982), and *S. variata* (Bock, 1982) which were added to the *brunnea* group in 1982. Due to the limited materials, the subgroup will not be discussed in this paper. The diagnosis of the *brunnea* group was revised by Bock (1982) as following: arista exceptionally large, fan-like, with curved rays; carina large; rather large species; prescutellar bristles weak.

In the present study, seven new species from East Asian are described, and three known species are redescribed. DNA barcoding was conducted to evaluate morphological delimitation for the *brunnea* group, and for this, a total of 44 *COI* (mitochondrial cytochrome c oxidase I) gene sequences of the above-mentioned ten species mentioned above are determined (Table 1).

**Table 1.** Specimens of the *brunnea* group used for DNA barcoding.

Species	Sex	BOLD Process ID	GenBank accession number	Collection site
*S. parabrunnea*	♂	BDORS034-15	KR070839	Menglun, Mengla, Yunnan, China
*S. pressobunnea* –1	♂	BDORS013-15	KR070841	Nonggang, Chongzuo, Guangxi, China
*S. pressobunnea* –2	♀	BDORS012-15	KR070840	Nonggang, Chongzuo, Guangxi, China
*S. scutellimargo* –1	♂	BDORS001-15	KR070847	Jianfengling, Ledong, Hainan, China
*S. scutellimargo* –2	♂	BDORS002-15	KR070854	Iriomote Is., Okinawa, Japan
*S. scutellimargo* –3	♀	BDORS003-15	KR070853	Longdong, Guangzhou, Guangdong, China
*S. scutellimargo* –4	♂	BDORS004-15	KR070852	Liuxihe, Conghua, Guangdong, China
*S. scutellimargo* –5	♂	BDORS005-15	KR070851	Wangtianshu, Mengla, Yunnan, China
*S. scutellimargo* –6	♂	BDORS006-15	KR070850	Wangtianshu, Mengla, Yunnan, China
*S. scutellimargo* –7	♂	BDORS008-15	KR070848	Wangtianshu, Mengla, Yunnan, China
*S. scutellimargo* –8	♂	BDORS007-15	KR070849	Menglun, Mengla, Yunnan, China
*S. scutellimargo* –9	♀	BDORM026-17	KY610504	Jianfengling, Ledong, Hainan, China
*S. maculata* sp. n. –1	♂	BDORS030-15	KR070820	Menglun, Mengla, Yunnan, China
*S. maculata* sp. n. –2	♂	BDORS031-15	KR070819	Menglun, Mengla, Yunnan, China
*S. maculata* sp. n. –3	♂	BDORS033-15	KR070821	Wangtianshu, Mengla, Yunnan, China
*S. maculata* sp. n. –4	♂	BDORS032-15	KR070818	Wangtianshu, Mengla, Yunnan, China
*S. maculata* sp. n. –5	♀	BDORM027-17	KY610505	Menglun, Mengla, Yunnan, China
*S. maculata* sp. n. –6	♀	BDORM028-17	KY610506	Wangtianshu, Mengla, Yunnan, China
*S. melanogaster* sp. n. –1	♂	BDORS020-15	KR070823	Baihualing, Baoshan, Yunnan, China
*S. melanogaster* sp. n. –2	♂	BDORS018-15	KR070824	Baihualing, Baoshan, Yunnan, China
*S. melanogaster* sp. n. –3	♂	BDORS017-15	KR070825	Hesong, Menghai, Yunnan, China
*S. melanogaster* sp. n. –4	♂	BDORS019-15	KR070822	Hesong, Menghai, Yunnan, China
*S. melanogaster* sp. n. –5	♂	BDORS021-15	KR070826	Menglun, Mengla, Yunnan, China
*S. melanogaster* sp. n. –6	♀	BDORM029-17	KY610507	Hesong, Menghai, Yunnan, China
*S. nigricostata* sp. n. –1	♂	BDORS022-15	KR070829	Baihualing, Baoshan, Yunnan, China
*S. nigricostata* sp. n. –2	♂	BDORS023-15	KR070827	Baihualing, Baoshan, Yunnan, China
*S. nigricostata* sp. n. –3	♂	BDORS024-15	KR070828	Wangtianshu, Mengla, Yunnan, China
*S. nigricostata* sp. n. –4	♀	BDORM030-17	KY610508	Baihualing, Baoshan, Yunnan, China
*S. nigripecta* sp. n. –1	♂	BDORS027-15	KR070831	Wangtianshu, Mengla, Yunnan, China
*S. nigripecta* sp. n. –2	♂	BDORS028-15	KR070832	Wangtianshu, Mengla, Yunnan, China
*S. nigripecta* sp. n. –3	♂	BDORS029-15	KR070830	Wangtianshu, Mengla, Yunnan, China
*S. obscurata* sp. n. –1	♂	BDORS035-15	KR070838	Wangtianshu, Mengla, Yunnan, China
*S. obscurata* sp. n. –2	♂	BDORS039-15	KR070834	Wangtianshu, Mengla, Yunnan, China
*S. obscurata* sp. n. –3	♂	BDORS037-15	KR070836	Menglun, Mengla, Yunnan, China
*S. obscurata* sp. n. –4	♀	BDORS038-15	KR070835	Menglun, Mengla, Yunnan, China
*S. obscurata* sp. n. –5	♂	BDORS036-15	KR070837	Menglun, Mengla, Yunnan, China
*S. obscurata* sp. n. –6	♂	BDORS040-15	KR070833	Hesong, Menghai, Yunnan, China
*S. protenipenis* sp. n. –1	♂	BDORS014-15	KR070843	Baihualing, Baoshan, Yunnan, China
*S. protenipenis* sp. n. –2	♂	BDORS016-15	KR070844	Hesong, Menghai, Yunnan, China
*S. protenipenis* sp. n. –3	♂	BDORS015-15	KR070842	Hesong, Menghai, Yunnan, China
*S. protenipenis* sp. n. –4	♀	BDORM031-17	KY610509	Hesong, Menghai, Yunnan, China
*S. rhina* sp. n. –1	♂	BDORS025-15	KR070845	Menglun, Mengla, Yunnan, China
*S. rhina* sp. n. –2	♂	BDORS026-15	KR070846	Baihualing, Baoshan, Yunnan, China
*S. rhina* sp. n. –3	♀	BDORM032-17	KY610510	Menglun, Mengla, Yunnan, China

## Materials and methods

### Specimens

The *brunnea* group flies were collected by net sweeping from tussocks and tree trunks near streams in forests. All the examined specimens were preserved in 75% ethanol. In the species descriptions, an asterisk * denotes a new record.

### Species identification

The specimens were first identified as of the *brunnea* group in light of morphology referring to Bock (1982) diagnosis of it. Then, they were examined for morphometric characters and detailed structures of terminalia, and sorted into putative species. For each of these putative species, representative specimens suitable for DNA sequencing were selected, considering also the numbers, geographical origins, and genders of available specimens. For each of the selected specimens, the total DNA was extracted from the abdominal tissue of samples after the dissection of the genitalia, using the TIANGEN DNA extraction kit following the recommended protocol. The PCR/sequencing primer pair was either that designed by He et al. (2009, 5'- CGCCT AAACT TCAGC CACTT -3'), or that by Folmer et al. (1994, 5’- GGTCAA CAAAT CATAA AGATA TTGG -3’, 5'-TAAAC TTCAG GGTGA CCAAA AAATC A-3'). The *COI* fragments were amplified using the cycle protocol as in Zhao et al. (2009).

All sequences generated determined in this study were submitted to BOLD (The Barcode of Life Data system) and GenBank (Table 1). A total of 44 *COI* sequences of the *brunnea* group were examined and aligned in MEGA 7.0 (Kumar et al. 2016). Then the inter- and intraspecific genetic distances were calculated for the species of the *brunnea* group using the p-distance model in MEGA 7.0. A NJ (Neighbor-joining) tree was constructed in MEGA 7.0 with p-distances.

In addition, we also conducted a character-based species delimitation. In the sequence alignment, sites being fixed within the focal species but differing from the remaining species were manually selected as diagnostic sites (i.e. “pure” diagnostics; Sarkar et al. 2002, Desalle et al. 2005) for each species. In this analysis, *S. latifasciaeformis* Duda, 1940 (GenBank accession number: GU597448) and *S. dorsocentralis* Okada, 1965 (GU597447), *S. puncticeps* Okada, 1956 (KJ841770, KJ841771) were used as the outgroups.

### Description of species

A Mshot Camera was used to microphotograph all the photographs, illustrations and line drawings were processed with the software Adobe Photoshop 7.0 and Easy Paint Tool SAI Ver.1.0.0. Zhang and Toda (1992) and Chen and Toda (2001) are followed for the definitions of measurements, indices and abbreviations.

The type specimens were deposited in Department of Entomology, South China Agricultural University, Guangzhou, China (**SCAU**).

## Results

The alignment of the 44 *COI* sequences spanned 664 nucleotide sites in length, with 202 variable sites, among which 177 were parsimony informative. The inter- and intraspecific p-distances between species of the *brunnea* group are given in Table 2. In most cases, the intraspecific p-distances in the *brunnea* group were less than 1%, while the largest intraspecific p-distance in the *brunnea* group was found in *S. melanogaster* sp. n. (= 2.7%). The interspecific p-distance ranged from 3.3% to 13.0%, while the smallest interspecific one was found between *S. maculata* sp. n. and *S. parabrunnea* sp. n.

**Figure 1.**
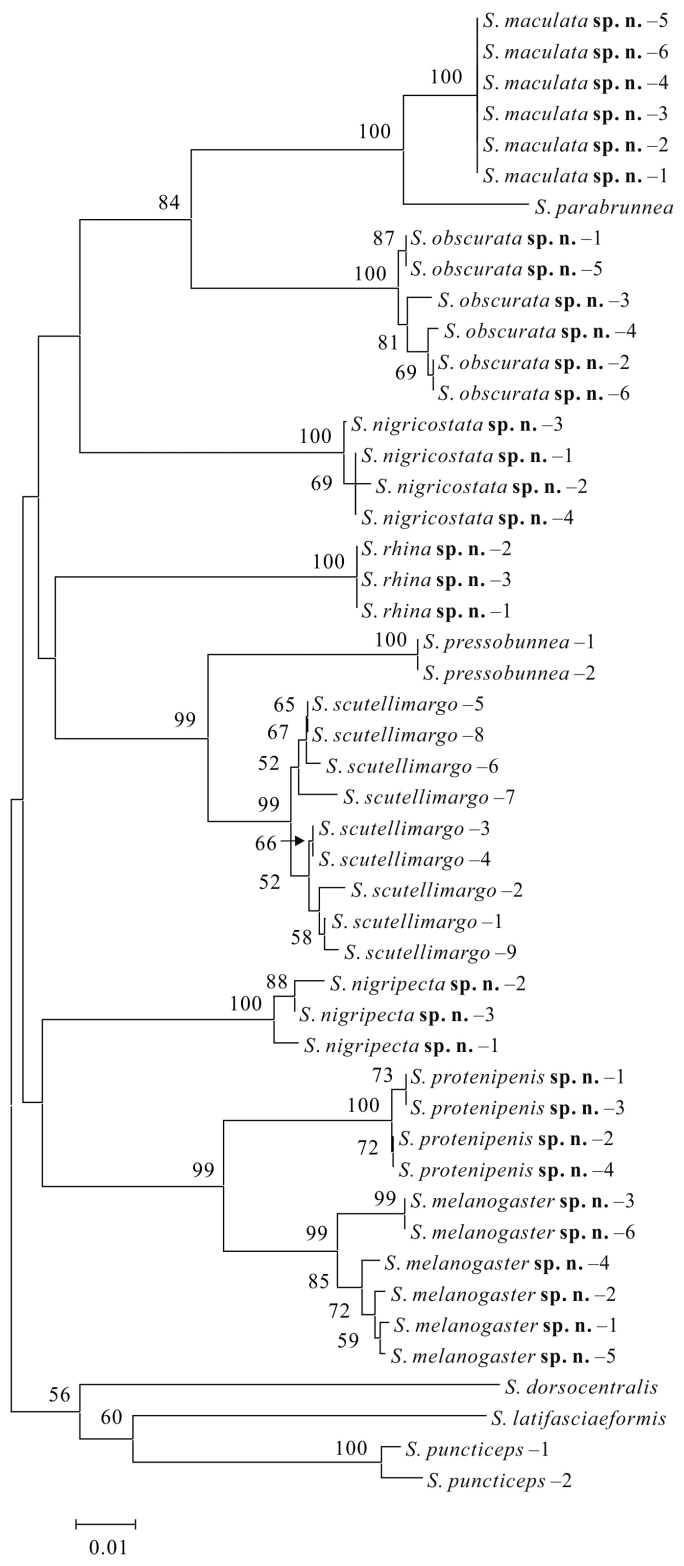
Neighbor-joining (NJ) tree of the *brunnea* group. The numbers around the nodes are bootstrap percentages (BP). BP values lower than 50 are not shown.

**Figure 2.**
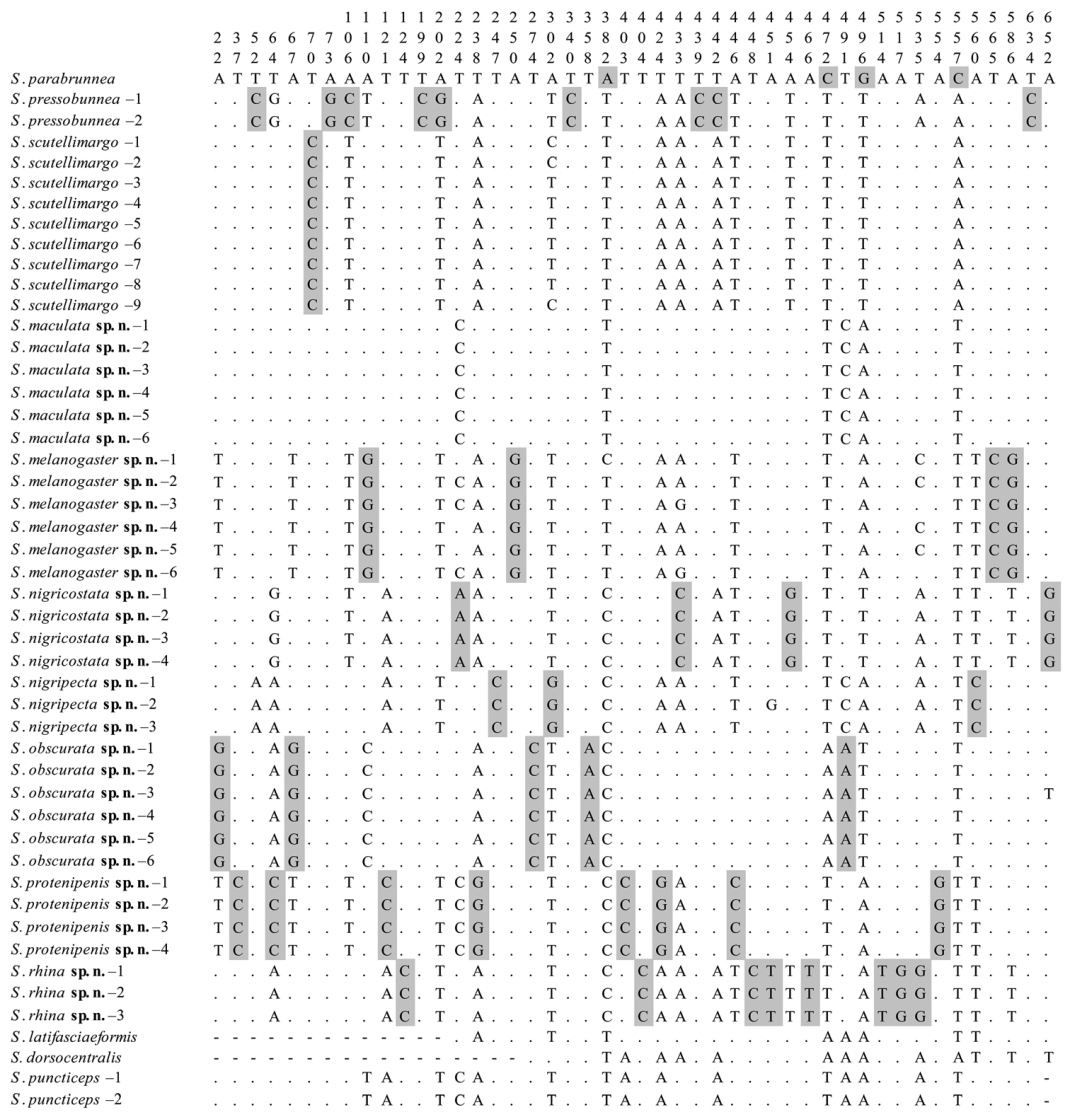
Diagnostic nucleotide sites in the alignment of *COI* sequences of the *brunnea* group. Numbers at the top show the positions of the sites in the *COI* alignment (660 bp in length). Shaded sites are diagnostic for each species. Hyphens (-) indicate missing data.

The NJ tree was shown in Fig. 1. In this tree, each morphologically recognized species was strongly supported [bootstraps percentage (BP) = 99 or 100, apart from *S. parabrunnea* with single specimen), and they formed a monophyletic group with respect to the outgroups (BP = 56). Fig. 2 shows nucleotides at the sites where “pure” diagnostics for any species of the *brunnea* group in this study. Except *S. maculata* sp. n., at least one diagnostic site was recognized for each species. For example, the site 124 is diagnostic for *S. rhina* sp. n.: this site has a fixed nucleotide status of C (Cytosine) in this species, but T (Thymidine) in the other species.

**Table 2.** Summary of intra- and interspecific genetic distances in the *brunnea* group.

Species	N	Intraspecific genetic distances	Interspecific genetic distances
		Min. / Max. / Mean ± SD	Min./ Max./ Mean ± SD
*S. parabrunnea*	1	NA	0.033/ 0.130/ 0.106 ± 0.031
*S. pressobunnea*	2	NA	0.053/ 0.125/ 0.103 ± 0.026
*S. scutellimargo*	9	0.002/ 0.024/ 0.009 ± 0.006	0.053/ 0.123/ 0.100 ± 0.014
*S. maculata* sp. n.	6	0.000/ 0.005/ 0.002 ± 0.002	0.033/ 0.125/ 0.107 ± 0.015
*S. melanogaster* sp. n.	6	0.000/ 0.027/ 0.017 ± 0.009	0.048/ 0.130/ 0.104 ± 0.019
*S. nigricostata* sp. n.	4	0.002/ 0.008/ 0.005 ± 0.002	0.087/ 0.123/ 0.103 ± 0.008
*S. nigripecta* sp. n.	3	0.003/ 0.008/ 0.005 ± 0.002	0.083/ 0.123/ 0.103 ± 0.009
*S. obscurata* sp. n.	6	0.000/ 0.009/ 0.005 ± 0.003	0.087/ 0.123/ 0.108 ± 0.010
*S. protenipenis* sp. n.	4	0.000/ 0.003/ 0.002 ± 0.001	0.048/ 0.128/ 0.100 ± 0.020
*S. rhina* sp. n.	3	0.000/ 0.003/ 0.002 ± 0.002	0.083/ 0.117/ 0.103 ± 0.013

**N** – the numbers of *COI* sequences involved in distance calculation; **Min.** – minimum; **Max.** – maximum; **SD** – standard deviation; **NA** – no applicable.

## Taxonomy

### *Scaptodrosophila brunnea* species group

Drosophila brunnea species group Tsacas & Chassagnard, 1976: 96; Bock, 1982: 72.

**Figure 3.**
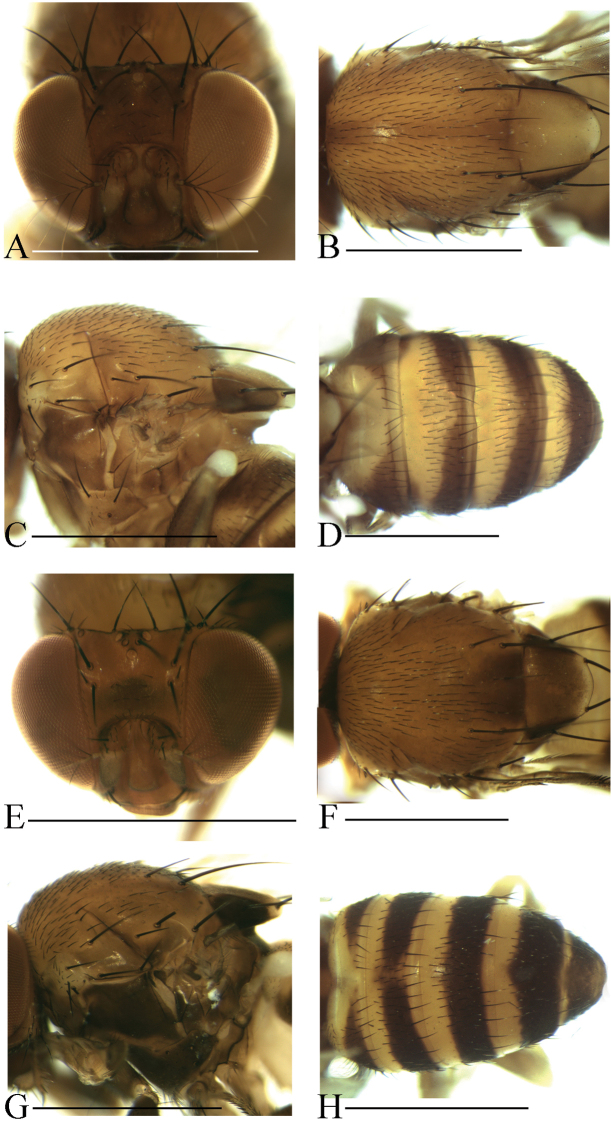
Head, mesonotum, scutellum, pleura and abdomen of male. **A–D** *S. parabrunnea* **E–H** *S. pressobrunnea*. Scale bars 1 mm.

**Figure 4.**
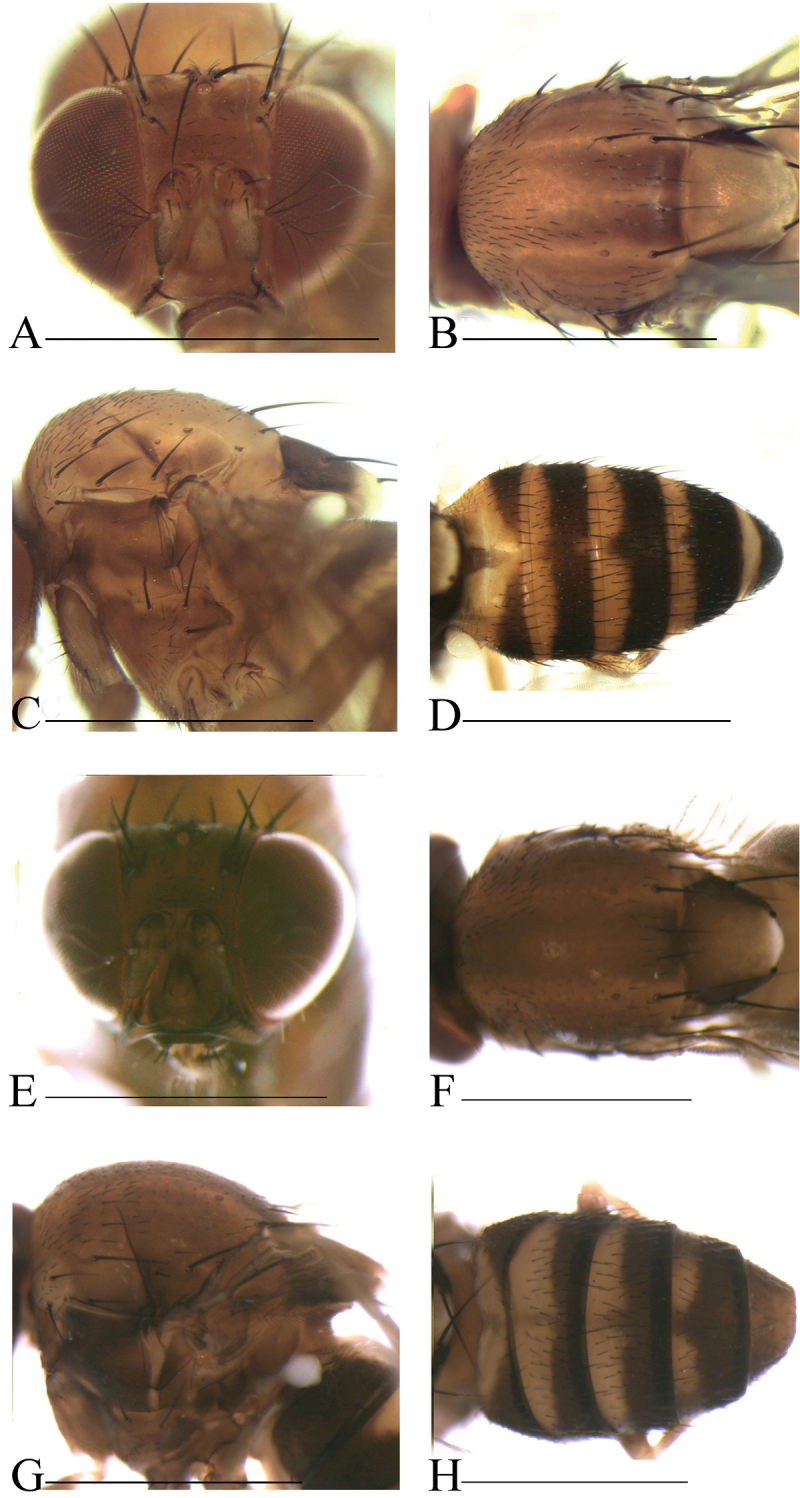
Head, mesonotum, scutellum, pleura and abdomen of male. **A–D** *S. scutellimargo* **E–H** *S. maculata* sp. n. Scale bars 1 mm.

**Figure 5.**
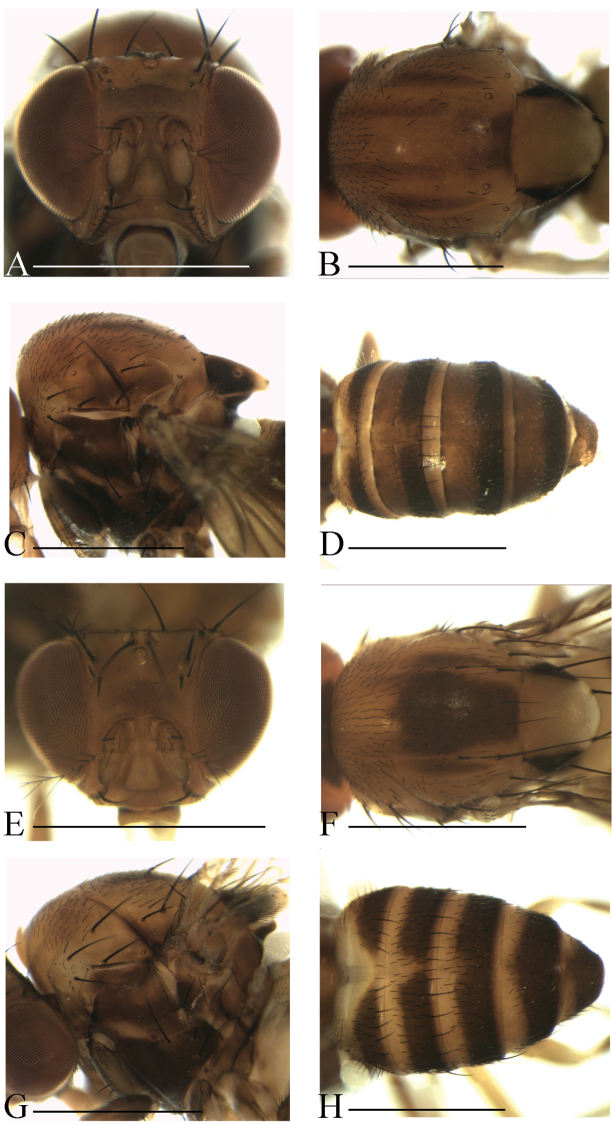
Head, mesonotum, scutellum, pleura and abdomen of male. **A–D** *S. melanogaster* sp. n. **E–H** *S. nigricostata* sp. n. Scale bars 1 mm.

**Figure 6.**
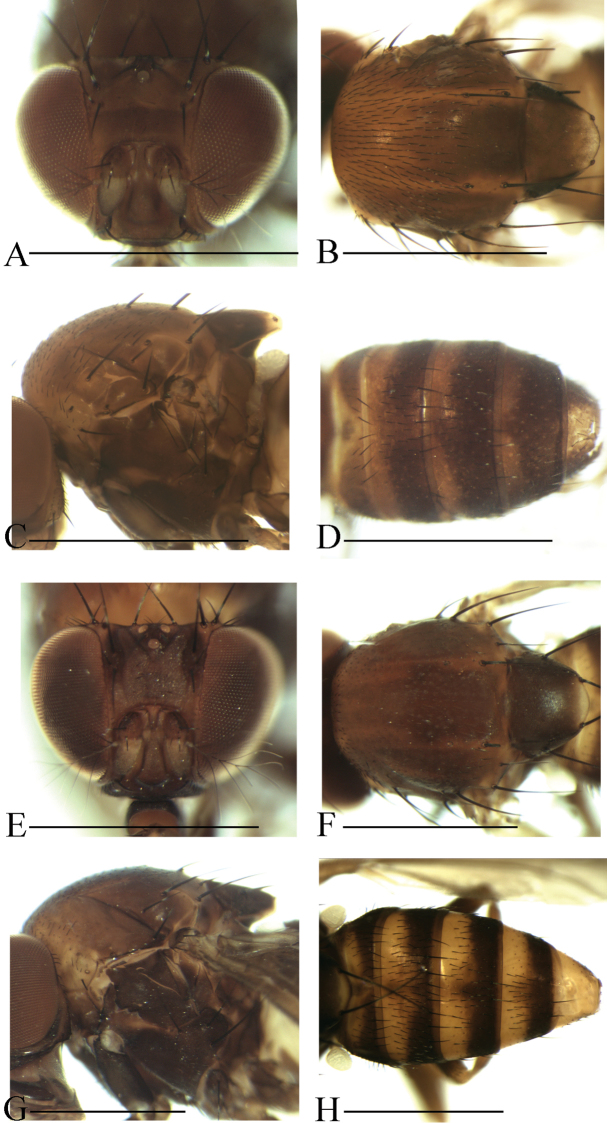
Head, mesonotum, scutellum, pleura and abdomen of male. **A–D** *S. nigripecta* sp. n. **E–H** *S. obscurata* sp. n. Scale bars 1 mm.

**Figure 7.**
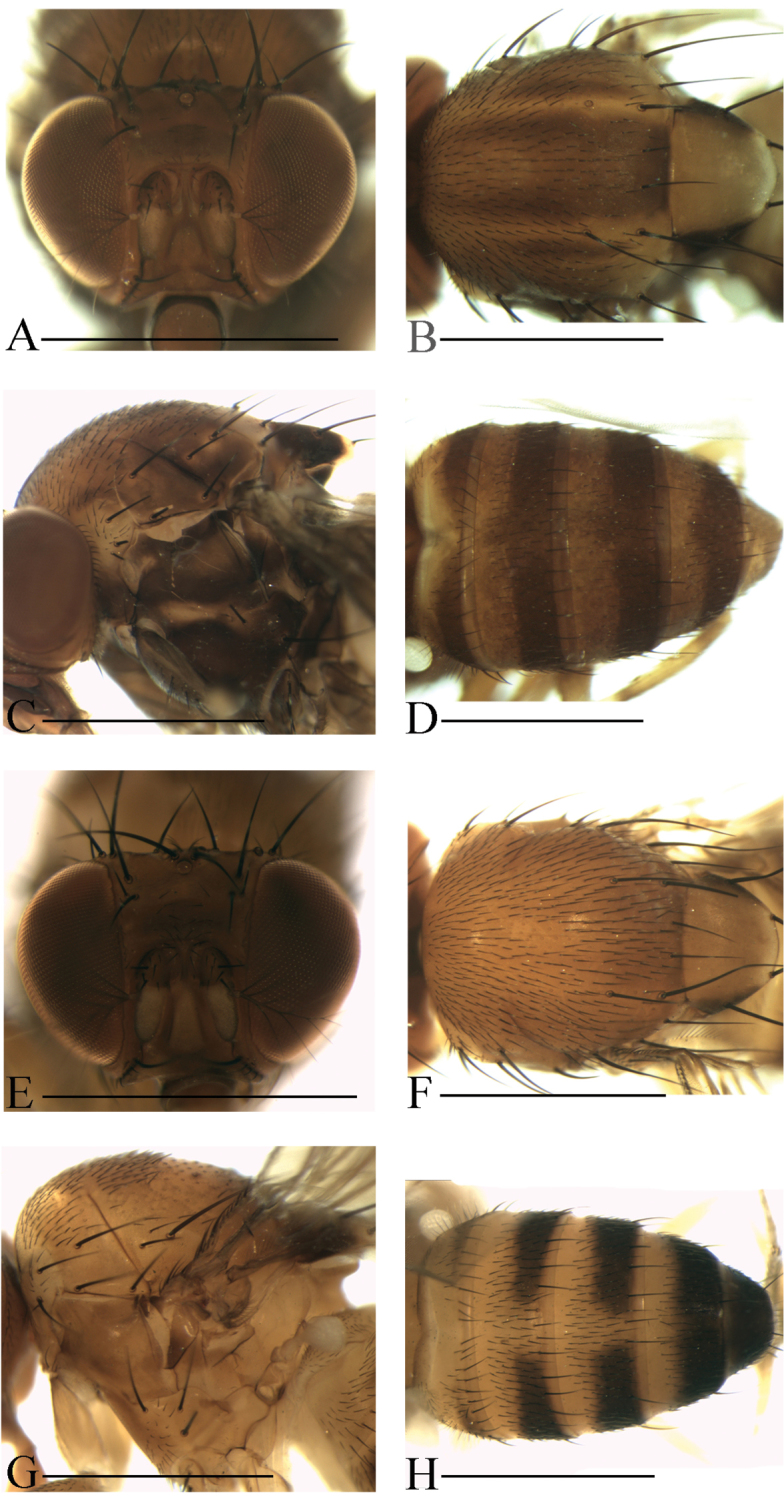
Head, mesonotum, scutellum, pleura and abdomen of male. **A–D** *S. protenipenis* sp. n. **E–H** *S. rhina* sp. n. Scale bars 1 mm.

**Diagnosis** (modified from Bock 1982). Arista exceptionally large, fan-like, with 4 (mostly) to 5 (occasionally) long, curved dorsal branches and 3 long, straight ventral branches in addition to terminal bifurcation (Figs 3–7A, E); facial carina large and prominent, as 2/5 length as face (Figs 3–7A, E).

**Description.** Male and female: *Head* (Figs 3–7A, E): eyes red to brownish red. Ocellar triangle yellowish brown to brown, mostly with 3 pairs of setae above ocellar setae. Frons nearly 1/3 width of head, with a few minute setulae medially. Anterior reclinate orbital setae usually outside and close to proclinate orbital setae; posterior reclinate orbital seta larger than others. Face usually yellowish brown to brown. Clypeus mostly yellowish brown to brown. Palpus usually yellowish brown. Vibrissa prominent; subvibrissal setae small. Gena and postgena narrow.

*Thorax* (Figs 3–7B, C, F, G): mesonotum yellowish brown to brown, usually with longitudinal stripe(s). Postpronotal lobe mostly yellowish, with 2–3 long setae and a few of shorter setae. Acrostichal setulae mostly in ca. 8–10 irregular rows. Prescutellar setae usually weak. Pleura mostly brown to dark brown. One small proepisternal seta. Katepisternal with three large setae and some small medially. Scutellum yellowish brown to brown, dark around basal scutellar setae, paler at tip. Wing hyaline, sometimes infuscate. Basal medial-cubital crossvein absent. R_4+5_ nearly parallel with M_1_ distally. Halter mostly white. Legs mostly yellowish brown.

*Abdomen* (Figs 3–7D, H): tergites yellow to yellowish brown anteromedially, with dark brown caudal bands.

**Figure 8.**
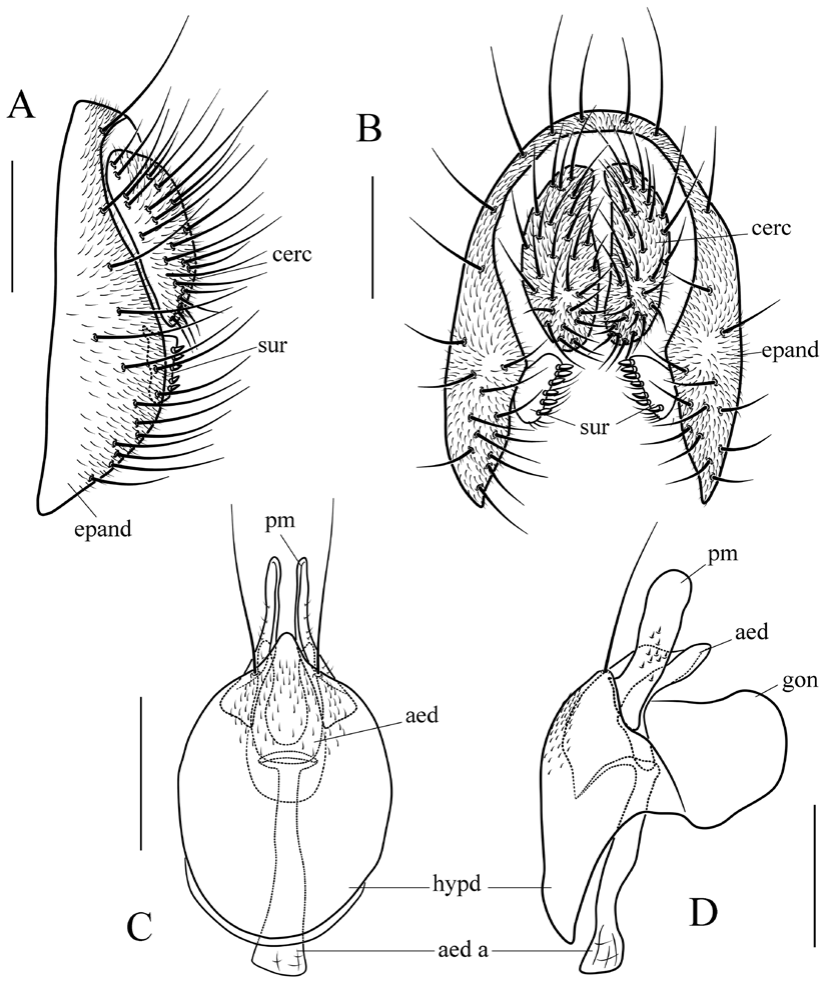
*Scaptodrosophila parabrunnea* (Tsacas & Chassagnard, 1976). **A, B** epandrium (epand), surstylus (sur) and cercus (cerc) (lateral and posterior views) **C, D** hypandrium (hypd), parameres (pm), gonopods (gon), aedeagus (aed) and aedeagal apodeme (aed a) (ventral and lateral views). Scale bars 0.1 mm.

**Figure 9.**
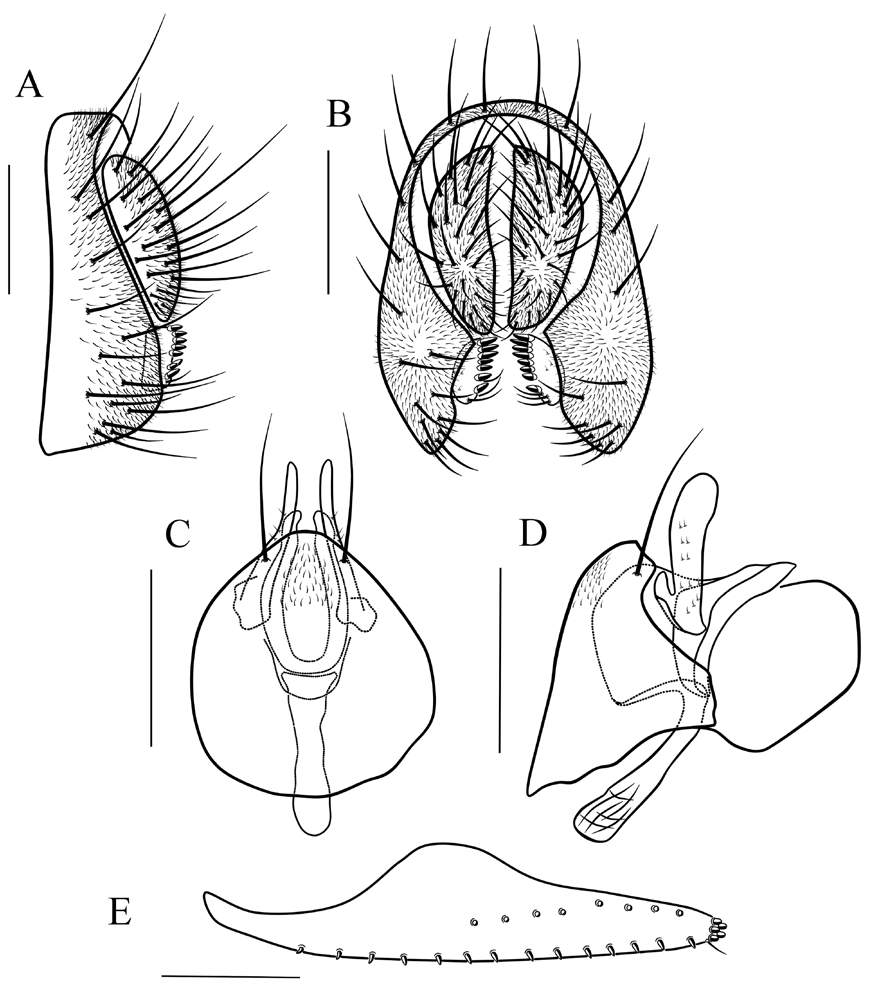
*Scaptodrosophila pressobrunnea* (Tsacas & Chassagnard, 1976). **A, B** epandrium, surstylus and cercus (lateral and posterior views) **C, D** hypandrium, parameres, gonopods, aedeagus and aedeagal apodeme (ventral and lateral views) **E** oviscapt (lateral view). Scale bars 0.1 mm.

**Figure 10.**
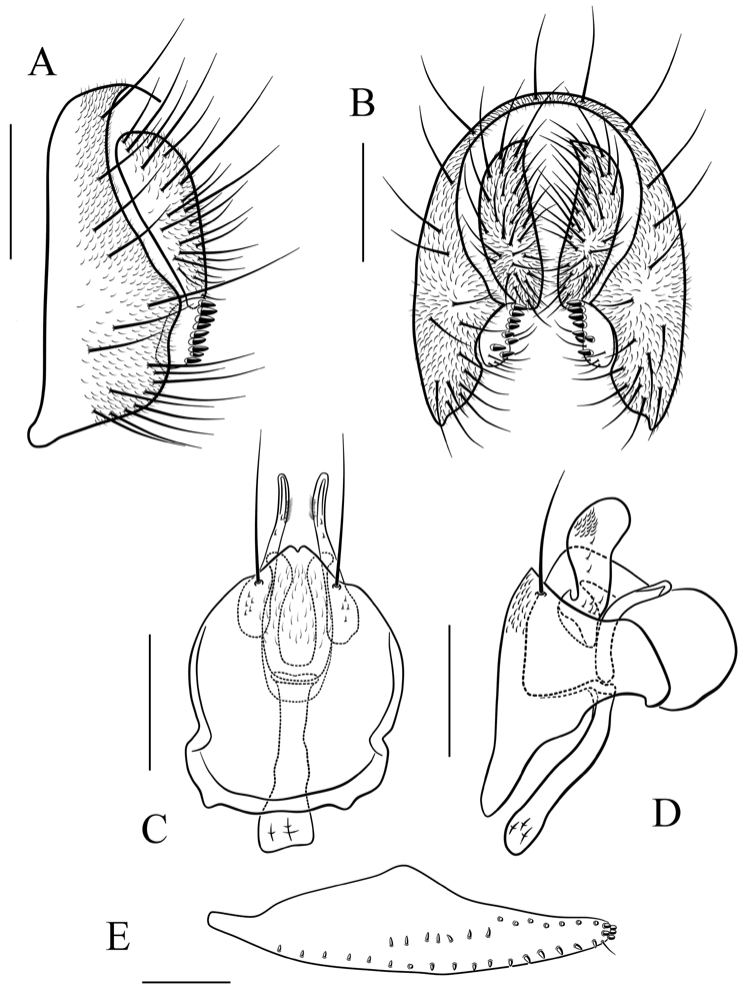
*Scaptodrosophila scutellimargo* (Duda, 1924). **A, B** epandrium, surstylus and cercus (lateral and posterior views) **C, D** hypandrium, parameres, gonopods, aedeagus and aedeagal apodeme (ventral and lateral views) **E** oviscapt (lateral view). Scale bars 0.1 mm.

**Figure 11.**
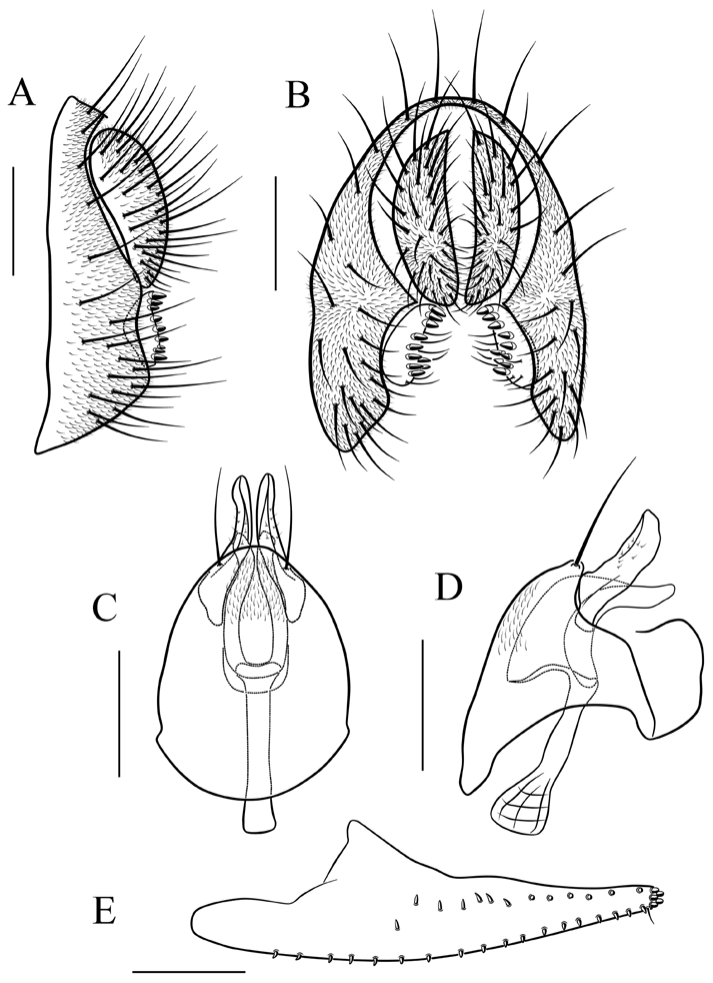
*Scaptodrosophila maculata* sp. n. **A, B** epandrium, surstylus and cercus (lateral and posterior views) **C, D** hypandrium, parameres, gonopods, aedeagus and aedeagal apodeme (ventral and lateral views) **E** oviscapt (lateral view). Scale bars 0.1 mm.

**Figure 12.**
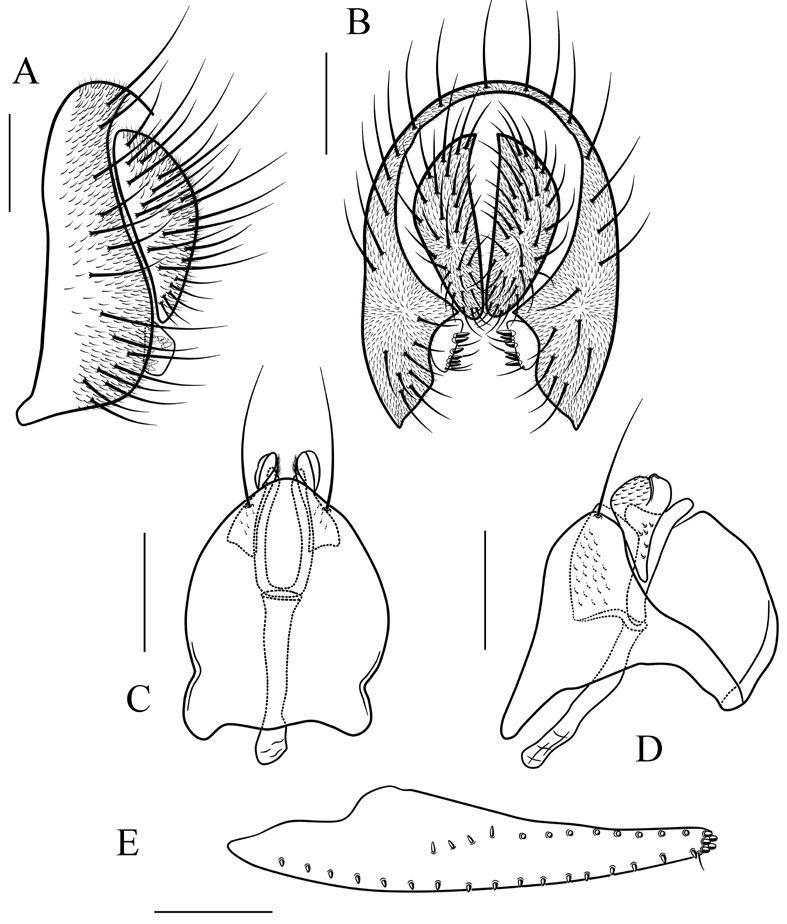
*Scaptodrosophila melanogaster* sp. n. **A, B** epandrium, surstylus and cercus (lateral and posterior views) **C, D** hypandrium, parameres, gonopods, aedeagus and aedeagal apodeme (ventral and lateral views) **E** oviscapt (lateral view). Scale bars 0.1 mm.

**Figure 13.**
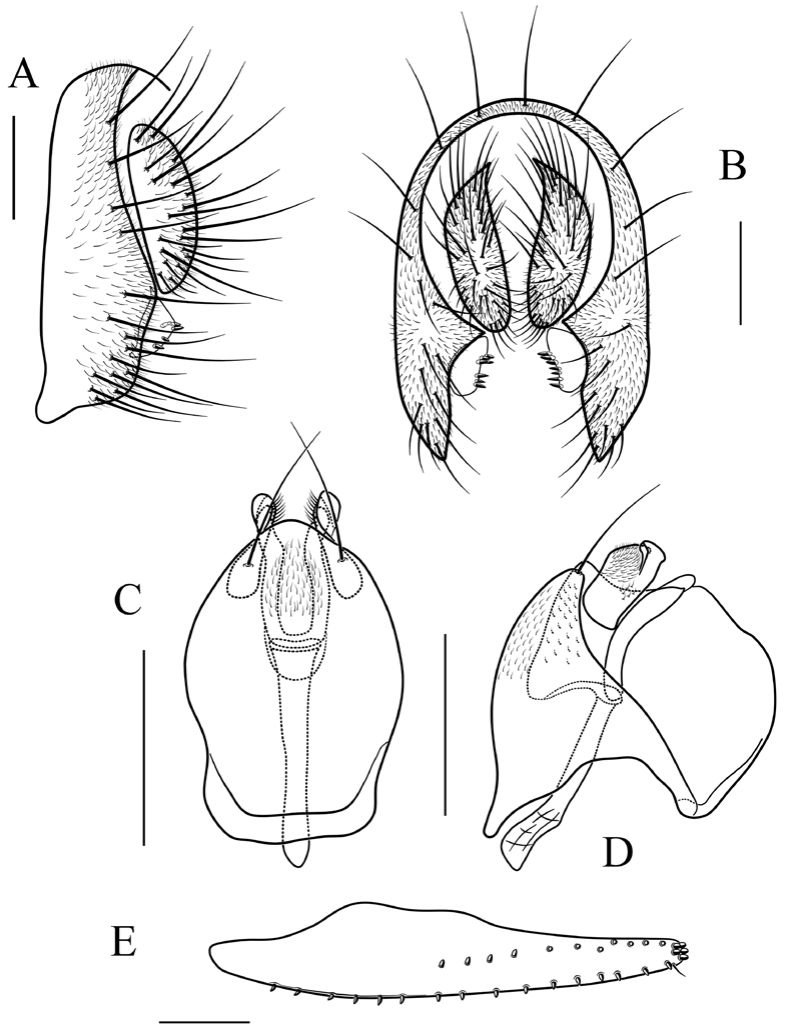
*Scaptodrosophila nigricostata* sp. n. **A, B** epandrium, surstylus and cercus (lateral and posterior views) **C, D** hypandrium, parameres, gonopods, aedeagus and aedeagal apodeme (ventral and lateral views) **E** oviscapt (lateral view). Scale bars 0.1 mm.

**Figure 14.**
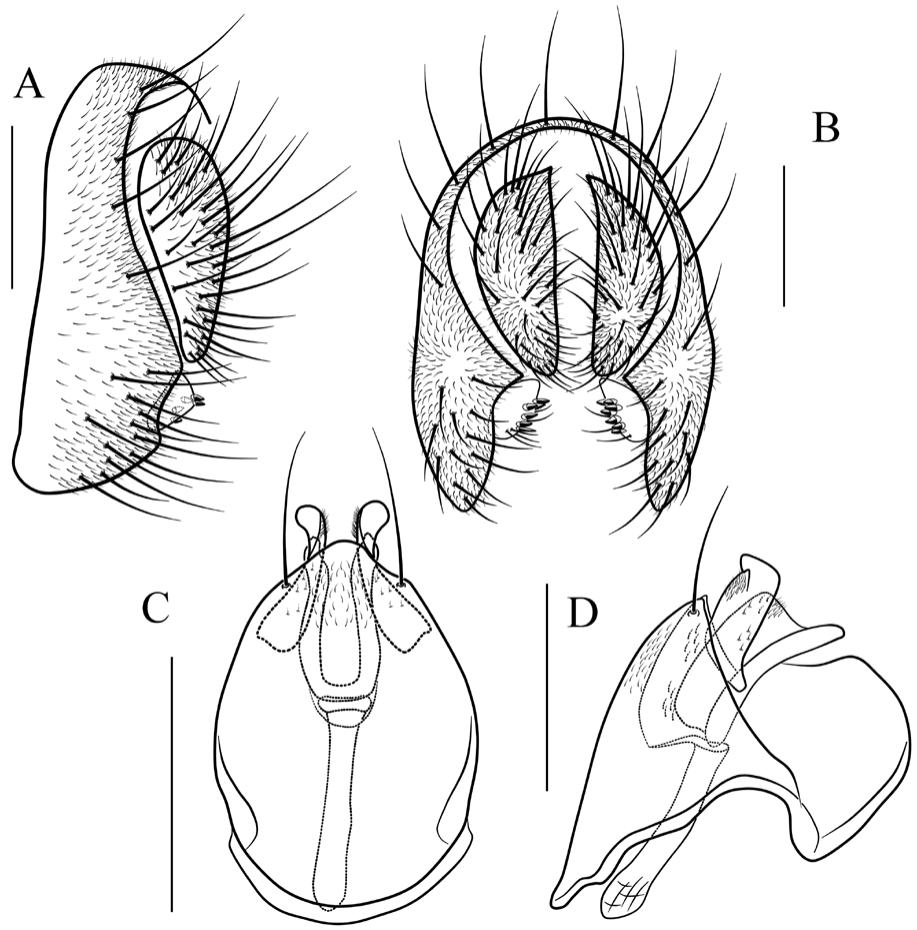
*Scaptodrosophila nigripecta* sp. n. **A, B** epandrium, surstylus and cercus (lateral and posterior views) **C, D** hypandrium, parameres, gonopods, aedeagus and aedeagal apodeme (ventral and lateral views). Scale bars 0.1 mm.

**Figure 15:**
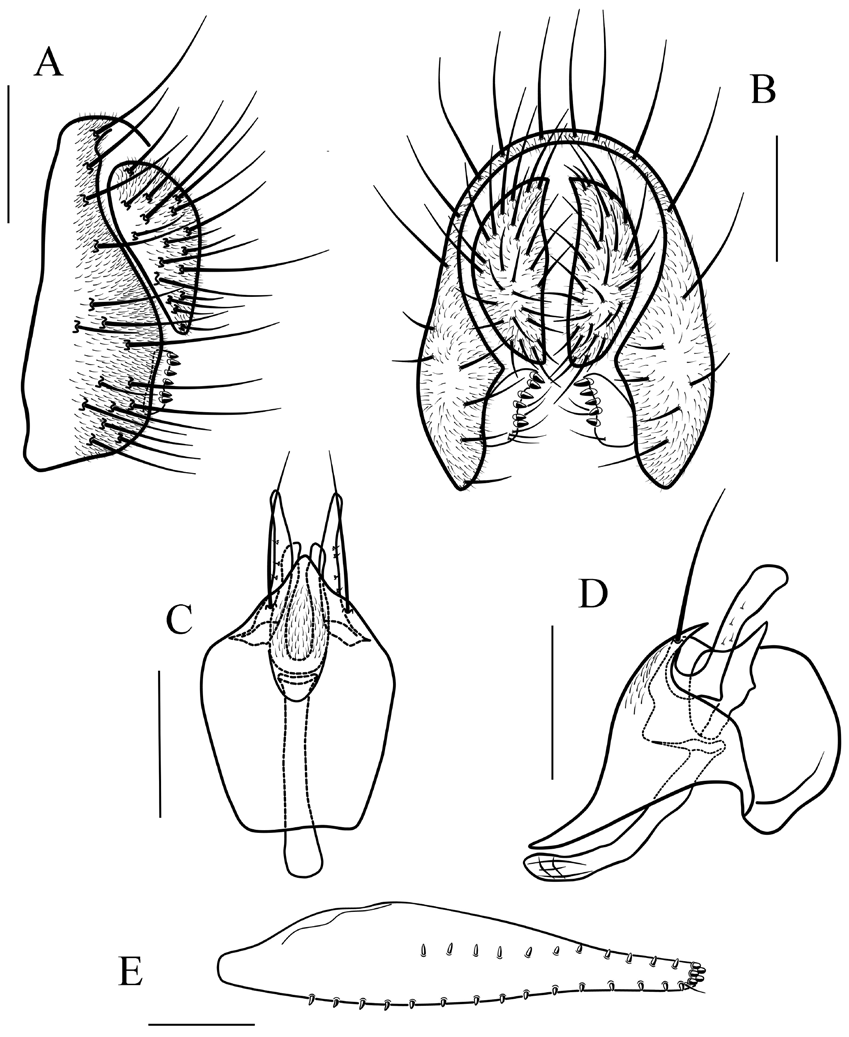
*Scaptodrosophila obscurata* sp. n. **A, B** epandrium, surstylus and cercus (lateral and posterior views) **C, D** hypandrium, parameres, gonopods, aedeagus and aedeagal apodeme (ventral and lateral views) **E** oviscapt (lateral view). Scale bars 0.1 mm.

**Figure 16.**
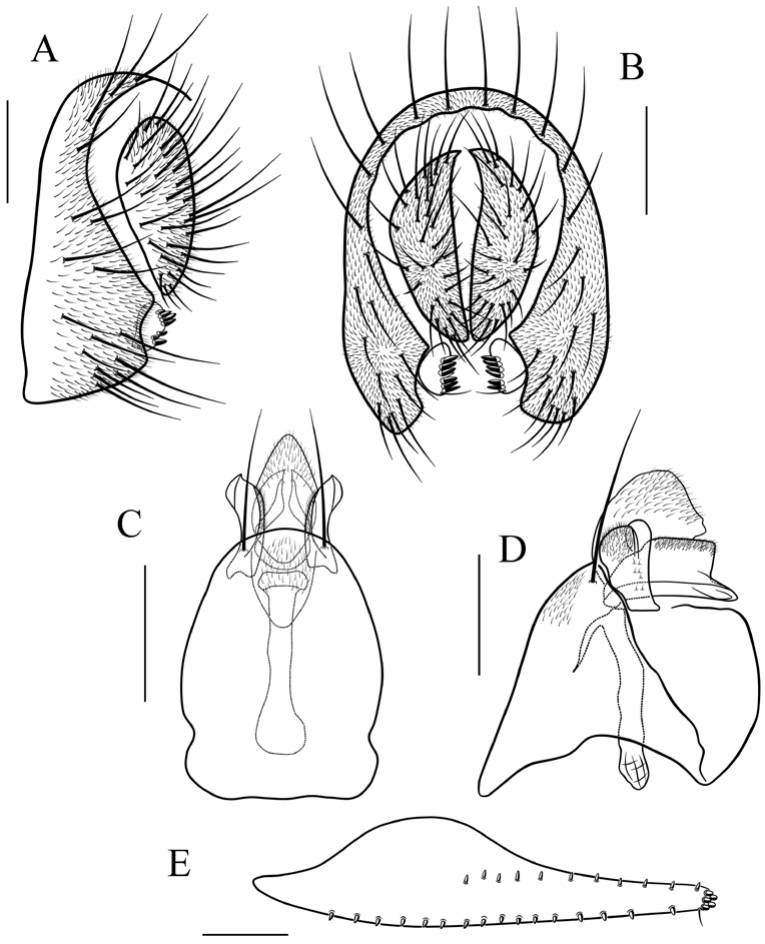
*Scaptodrosophila protenipenis* sp. n. **A, B** epandrium, surstylus and cercus (lateral and posterior views) **C, D** hypandrium, parameres, gonopods, aedeagus and aedeagal apodeme (ventral and lateral views) **E** oviscapt (lateral view). Scale bars 0.1 mm.

**Figure 17.**
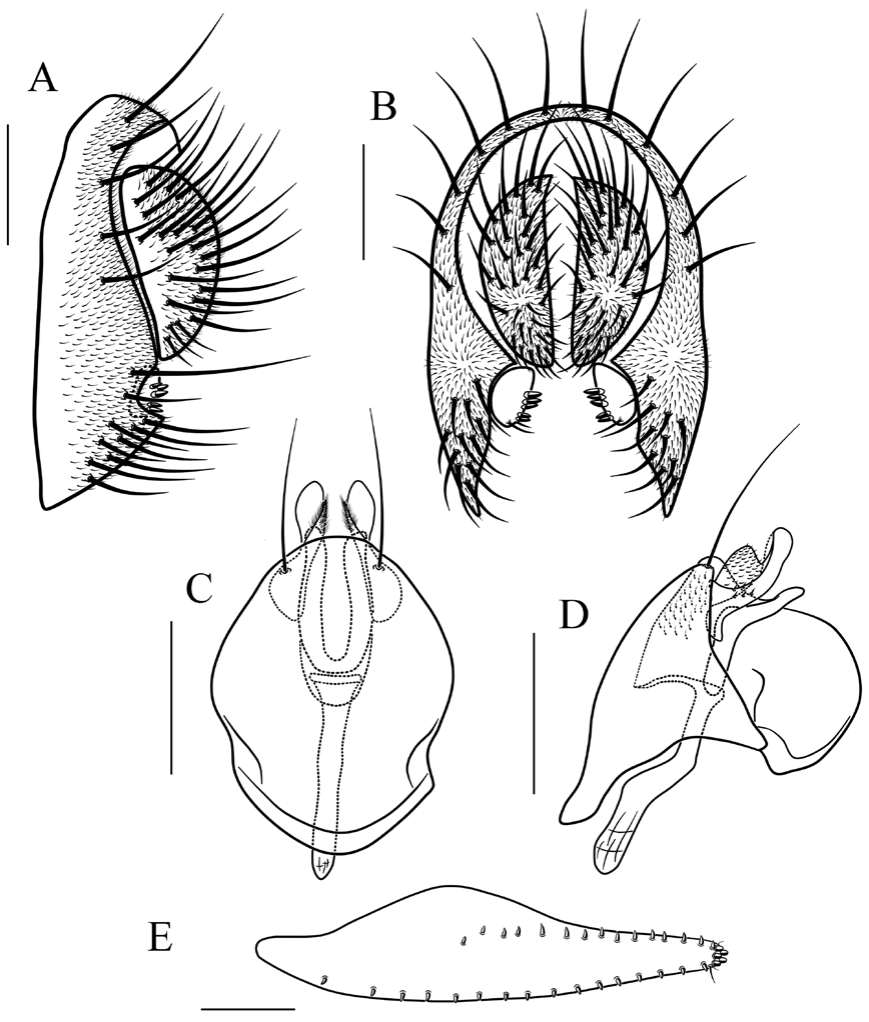
*Scaptodrosophila rhina* sp. n. **A, B** epandrium, surstylus and cercus (lateral and posterior views) **C, D** hypandrium, parameres, gonopods, aedeagus and aedeagal apodeme (ventral and lateral views) **E** oviscapt (lateral view). Scale bars 0.1 mm.

*Male terminalia* (Figs 8–17A–D): epandrium usually pubescent, with several setae around anteroventral corner to posterior margin. Surstylus with several peg-like prensisetae apically, several setae on outer and inner surfaces. Cercus separated from epandrium, pubescent and setigerous. Hypandrium pale brown, usually with paramedian setae. Paramere with several sensilla. Gonopods fused with each other, broadened to hood-shaped. Aedeagus bilobed subbasally.

*Female terminalia* (Figs 9–13, 15–17E): oviscapt valve long, mostly yellowish brown, usually with one subapical trichoid ovisensillum and approximately 16, 12, 5 peg-like ovisensilla per side on ventral, dorsal and apical margins, respectively.

In the following individual species descriptions, only characters that depart from the above universal characters are provided for brevity.

#### Scaptodrosophila parabrunnea

Taxon classificationAnimaliaORDOFAMILIA

(Tsacas & Chassagnard, 1976)

Figs 3A–D , 8

Drosophila parabrunnea Tsacas & Chassagnard, 1976: 92.

##### Specimen examined.

CHINA: 1♂ (SCAU, No. 128342), Menglun, Mengla, Yunnan, 21°55'N, 101°16'E, alt. 570 m, 3–4.xi.2001, JJ Gao.

##### Diagnosis.

This species is very similar to *S. maculata* sp. n. in the patterns of abdominal tergites (Fig. 3B) and aedeagus curved dorsally in lateral view (Fig. 8D), but it can be distinguished from the latter by having the paramere apically round in lateral view (Fig. 8D); gonopods dorsally expanded in lateral view (Fig. 8D); see under that species.

##### Description.

Male and female: *Head* (Fig. 3A): frons yellowish to brown. Pedicel brownish; first flagellomere yellowish brown. Facial carina brown, short, as 1/3 length as face.

*Thorax* (Fig. 3B, C): mesonotum yellowish brown, with a brown longitudinal stripe medially. Acrostichal setulae in ca. 8–10 irregular rows. Scutellum yellow, dark brown near basal scutellar setae, pale at tip. Pleura brownish.

*Abdomen* (Fig. 3D): tergites II to V yellow, with dark brown caudal bands, the caudal bands on tergites II and III narrowed medially; tergite VI entirely dark brown.

*Male terminalia* (Fig. 8): epandrium with ca. 16 setae near posterior and ventral margins per side. Surstylus with 6–7 peg-like prensisetae. Hypandrium with a pair of paramedian setae and pubescence basomedially. Paramere with ten sensilla medially. Aedeagus lacking pubescence.

*Measurements* (in mm). BL = 3.07, ThL =1.47, WL =3.13, WW =1.13.

*Indices*: arb = 4/3, avd = 0.90, adf = 2.50, flw = 2.00, FW/HW = 0.43, ch/o = 0.05, prorb = 0.50, rcorb = 0.27, vb = 0.33, dcl = 0.45, presctl = 0.29, sctl = 0.80, sterno = 0.71, orbito = 0.50, dcp = 0.39, sctlp = 0.75, C = 2.05, 4c = 0.95, 4v = 2.00, 5x = 1.67, ac = 2.00, M = 0.52, C3F = 0.83.

##### Distribution.

China* (Yunnan), Indonesia (Java, Sumatra).

#### Scaptodrosophila pressobrunnea

Taxon classificationAnimaliaORDOFAMILIA

(Tsacas & Chassagnard, 1976)

Figs 3E–H , 9

Drosophila pressobrunnea Tsacas & Chassagnard, 1976: 93.

##### Specimens examined.

CHINA: 1♂, 1♀ (SCAU, Nos 128246, 47), Nonggang, Chongzuo, Guangxi, 25°00'N, 106°51'E, alt. 230 m, 21–24.viii.2004, HW Chen.

##### Diagnosis.

This species is very similar to *S. scutellimargo* in the patterns of abdominal tergites (Fig. 3H) and aedeagus curved dorsal (Fig. 9D), but can be distinguished from the latter by having the paramere slightly broadened distally in lateral view (Fig. 9D); gonopods elliptically expanded dorsally in lateral view (Fig. 9D); see under that species.

##### Description.

Male and female: *Head* (Fig. 3E): frons yellowish brown with a brown band anteriorly. Pedicel brownish; first flagellomere yellowish. Facial carina yellowish brown.

*Thorax* (Fig. 3F, G): mesonotum yellowish brown, with a brown longitudinal stripe on 1/3 posterior. Acrostichal setulae in ca. 8–10 irregular rows. Scutellum brownish, dark brown near basal scutellar setae, pale at tip. Pleura dark brown.

*Abdomen* (Fig. 3H): tergites II to V yellow, with dark brown caudal bands, the caudal band on tergite II narrowed medially; tergite VI entirely dark brown.

*Male terminalia* (Fig. 9A–D): epandrium with ca. 15 setae near posterior and ventral margins per side. Surstylus with 6–7 peg-like prensisetae. Hypandrium with a pair of paramedian setae and pubescence basomedially. Paramere with 12 sensilla, and a small projection basally. Aedeagus lacking pubescence.

*Female terminalia* (Fig. 9E): oviscapt with one subapical trichoid ovisensillum, 14, 8 and 5 peg-like ovisensilla per side on ventral, dorsal and apical margins, respectively.

*Measurements* (range in 1♂, 1♀, in mm): BL = (2.89, 2.98), ThL = (1.29, 1.16), WL = (2.62, 2.36), WW = (1.02, 0.93).

*Indices*: arb = 4/3, avd = 0.89–0.94, adf = 3.60, flw = 2.00, FW/HW = 0.40–0.42, ch/o = 0.08–0.09, prorb = 0.65–0.68, rcorb = 0.26–0.35, vb = 0.83–1.00, dcl = 0.59, presctl = 0.38–0.45, sctl = 0.97–1.07, sterno = 0.68–0.70, orbito = 0.44–0.50, dcp = 0.38–0.44, sctlp = 0.79–1.10, C = 1.83–1.89, 4c = 1.33–1.40, 4v = 2.41–2.52, 5x = 1.80–1.90, ac = 3.27–3.50, M = 0.70–0.72, C3F = 0.88–0.90.

##### Distribution.

China* (Guangxi), India, Indonesia (Sumatra).

#### Scaptodrosophila scutellimargo

Taxon classificationAnimaliaORDOFAMILIA

(Duda, 1924)

Figs 4A–D , 10

Drosophila scutellimargo Duda, 1924: 243; Tsacas & Chassagnard, 1976: 92.

##### Specimens examined.

CHINA: 4♀ (SCAU, Nos 128204–07), Longdong, Guangzhou, Guangdong, 12°19'N, 113°21'E, alt. 200 m, 1.v.2007, HW Chen; 2♀ (SCAU, Nos 128378–79), Tianluhu Park, Guangzhou, Guangdong, 23°13'N, 113°09'E, alt. 240 m, 6.ix.2015, YL Wang; 1♂ (SCAU, No. 128208), Liuxihe, Conghua, Guangdong, 23°26'N, 113°30'E, alt. 200 m, 12.v.2010, XY Xu; 9♂, 3♀ (SCAU, Nos 128189–200), Jianfengling, Ledong, Hainan, 18°41'N, 108°52'E, alt. 680–820 m, 23.iv.2007, XP Chen, JJ Gao; 2♂, 3♀ (SCAU, Nos 128377–81), Mulun, Huangjiang, Guangxi, 25°09'N, 108°01'E, alt. 449 m, 19.ix.2015, 26.vii.2015, YQ Liu; 1♂, 2♀ (SCAU, Nos 128382–84), Weng’ang, Libo, Guizhou, 25°13'N, 107°56'E, alt. 754 m, 16.ix.2015, L Zhu; 6♂, 7♀ (SCAU, Nos 128229–40, 128370), Menglun, Mengla, Yunnan, 24°41'N, 101°25'E, alt. 680 m, 17.iv.2007, HW Chen, JJ Gao; 10♂, 10♀ (SCAU, Nos 128209–28), Wangtianshu, Mengla, Yunnan, 24°41'N, 101°25'E, alt. 680 m, 22–25.iv.2007, 9.x.2012, HW Chen, JJ Gao. JAPAN: 1♂, 2♀ (SCAU, Nos 128201–03), Iriomote Island, Okinawa, 24°32'N, 123°88'E, alt. 150 m, 12.v.2001, HW Chen.

##### Diagnosis.

Paramere distally broadened and pubescent in lateral view (Fig. 10D); gonopods roundly expanded dorsally in lateral view (Fig. 10D). The 5.3% interspecific genetic distance to *S. scutellimargo* is one of the smallest interspecific distances ascertained within this group (Table 2).

##### Description.

Male and female: *Head* (Fig. 4A): frons yellowish brown. Pedicel brownish; first flagellomere yellowish. Facial carina yellowish, short, as 1/3 length as face.

*Thorax* (Fig. 4B, C): mesonotum brown, with three yellowish brown longitudinal stripes. Acrostichal setulae in ca. 8–10 irregular rows. Scutellum yellowish, dark brown near basal scutellar setae, pale at tip. Pleura brownish to brown.

*Abdomen* (Fig. 4D): all tergites yellow with dark brown caudal bands, the caudal band on tergite II narrowed medially.

*Male terminalia* (Fig. 10A–D): epandrium with ca. 16 setae near posterior and ventral margins per side. Surstylus with eight peg-like prensisetae. Hypandrium with a pair of paramedian setae and pubescence basomedially. Paramere with eight sensilla medially distally. Aedeagus lacking pubescence.

*Female terminalia* (Fig. 10E): oviscapt with one subapical trichoid ovisensillum, 17, 14 and 5 peg-like ovisensilla per side on ventral, dorsal and apical margins, respectively.

*Measurements* (range in 7♂, 3♀, in mm): BL = (2.73–3.20, 3.07–3.33), ThL = (1.29–1.42, 1.07–1.47), WL = (2.53–2.98, 2.87–3.07), WW = (0.93–1.07, 1.00–1.20).

*Indices*: arb = 4/3, avd = 0.94–1.06, adf = 0.40–0.80, flw = 2.00–2.50, FW/HW = 0.38–0.42, ch/o = 0.06–0.09, prorb = 0.60–0.74, rcorb = 0.20–0.33, vb = 0.83–1.25, dcl = 0.51–0.59, presctl = 0.31–0.44, sctl = 0.87–1.03, sterno = 0.57–0.69, orbito = 0.40–0.60, dcp = 0.36–0.48, sctlp = 0.86–1.00, C = 1.97–2.20, 4c = 1.00–1.20, 4v = 2.00–2.27, 5x = 1.92–2.20, ac = 2.27–2.92, M = 0.62–0.77, C3F = 0.90–0.94.

##### Distribution.

China (Taiwan, Guangdong, Hainan*, Guangxi*, Guizhou*, Yunnan), Japan* (Ryukyu Is.).

#### Scaptodrosophila maculata sp. n.

Taxon classificationAnimaliaORDOFAMILIA

http://zoobank.org/2D412DD1-B660-45B4-AF54-F11970EB111D

Figs 4E–H , 11

##### Type material.

Holotype ♂ (SCAU, No. 128318): CHINA: Wangtianshu, Mengla, Yunnan, 21°47'N, 101°63'E, alt. 760 m, 23.iv.2007, HW Chen. Paratypes: CHINA: 2♂, 6♀ (SCAU, Nos 128319–26), HW Chen, JJ Gao, same data as holotype; 5♂, 8♀ (SCAU, Nos 128329–39, 128371, 72), Wangtianshu, Mengla, Yunnan, 21°47'N, 101°63'E, alt. 760 m, 9.v.2012, HW Chen, JJ Gao; 2♂ (SCAU, Nos 128327–28), Menglun, Mengla, Yunnan, 21°55'N, 101°16'E, alt. 570 m, 3–4.xi.2001, HW Chen.

##### Diagnosis.

Paramere distally curved ventrally, slightly acute apically (Fig. 11D); gonopods not expanded dorsal in lateral view (Fig. 11D). The 3.3% interspecific genetic distance to *S. parabrunnea* is one of the smallest interspecific distances ascertained within this group (Table 2).

##### Description.

Male and female: *Head* (Fig. 4E): frons brownish. Pedicel yellowish brown to brown; first flagellomere yellowish. Facial carina nose-like, brown.

*Thorax* (Fig. 4F, G): mesonotum brown, with two yellowish brown longitudinal stripes submedially. Acrostichal setulae in ca. 8–10 irregular rows. Scutellum brownish, dark brown near basal scutellar setae, pale at tip. Pleura brownish to dark brown.

*Abdomen* (Fig. 4H): tergites II to V yellow with dark brown caudal bands, the caudal bands on tergites II and III narrowed medially; tergite VI dark brown.

*Male terminalia* (Fig. 11A–D): epandrium with ca. 19 setae near posterior and ventral margins per side. Surstylus with ten peg-like prensisetae (Fig. 11B). Hypandrium with a pair of paramedian setae and pubescence medially). Paramere with eight sensilla distally. Aedeagus lacking pubescence.

*Female terminalia* (Fig. 11E): oviscapt with one subapical trichoid ovisensillum, 18, 13 and 5 peg-like ovisensilla per side on ventral, dorsal and apical margins, respectively.

*Measurements* [holotype (paratypes range in 4♂, 5♀), in mm]. BL = 3.91 (3.16–3.96, 2.76–3.60), ThL = 1.82 (1.33–1.64, 1.29–1.64), WL = 3.20 (2.49–3.51, 2.40–3.16), WW = 1.38 (1.07–1.11, 1.11–1.33).

*Indices*: arb = 4/3 (4/3), avd = 1.22 (0.75–1.13), adf = 3.00 (2.67–4.00), flw = 1.67 (1.33–2.50), FW/HW = 0.57 (0.31–0.49), ch/o = 0.10 (0.10–0.18), prorb = 0.79 (0.55–0.78), rcorb = 0.57 (0.23–0.44), vb = 1.00 (0.50–1.00), dcl = 0.83 (0.57–0.95), presctl = 0.50 (0.43–0.52), sctl = 0.91 (0.91–1.29), sterno = 0.77 (0.64–0.92), orbito = 0.50 (0.50–1.00), dcp = 0.53 (0.44–0.53), sctlp = 0.88 (0.83–1.17), C = 2.12 (1.95–2.63), 4c = 1.13 (0.91–1.24), 4v = 2.17 (2.09–2.56), 5x = 1.88 (1.43–2.71), ac = 2.60 (2.10–2.93), M = 0.65 (0.59–0.91), C3F = 0.92 (0.82–0.96).

##### Etymology.

From the Latin word “*maculatus*” (= spotted), referring to the mesonotum with dark patch.

##### Distribution.

China (Yunnan).

#### Scaptodrosophila melanogaster sp. n.

Taxon classificationAnimaliaORDOFAMILIA

http://zoobank.org/EDD6A14D-2C1D-453F-AE83-13F275EC9E07

Figs 5A–D , 12

##### Type material.

Holotype ♂ (SCAU, No. 128297): CHINA: Baihualing, Baoshan, Yunnan, 25°17'N, 98°48'E, alt. 1400 m, 7.vi.2011, HW Chen. Paratypes: CHINA: 6♂, 10♀ (SCAU, Nos 128298–313), HW Chen, JJ Gao, same data as holotype; 2♂, 1♀ (SCAU, Nos 128314–15, 128373), Hesong, Menghai, Yunnan, 21°50'N, 100°05'E, alt. 1940 m, 16.iv.2010, 6.v.2012, HW Chen, JM Lu; 1♂ (SCAU, No. 128317), Menglun, Mengla, Yunnan, alt. 570 m, 3, 4.xi.2001, HW Chen.

##### Diagnosis.

This species is similar to *S. rhina* sp. n. in the male terminalia, but can be distinguished from the latter by having the paramere expanded and not divided distally in lateral view (Fig. 12D), the aedeagus distally protruded ventrally in lateral view (Fig. 12D), the mesonotum yellowish brown, with four brown longitudinal stripes sublaterally (Fig. 5B); see under that species.

##### Description.

Male and female: *Head* (Fig. 5A): frons yellowish brown with a brown band anteriorly. Pedicel yellowish brown; first flagellomere yellowish. Facial carina yellowish, short, as 1/3 length as face.

*Thorax* (Fig. 5B, C): acrostichal setulae in ca. 8–10 irregular rows. Scutellum yellowish brown, dark brown near basal scutellar setae, pale at tip. Pleura dark brown.

*Abdomen* (Fig. 5D): tergites II to V brown with dark brown caudal bands, the caudal band on tergite II interrupted medially; tergite VI brown.

*Male terminalia* (Fig. 12A–D): epandrium with ca. 16 setae near posterior and ventral margins per side. Surstylus with 6–7 peg-like prensisetae. Hypandrium with a pair of paramedian setae, lacking pubescence. Paramere with six sensilla subbasally and pubescence distally. Aedeagus lacking pubescence.

*Female terminalia* (Fig. 12E): oviscapt with one subapical trichoid ovisensillum, 17, 12 and 5 peg-like ovisensilla per side on ventral, dorsal and apical margins, respectively.

*Measurements* [holotype (paratypes range in 4♂, 5♀), in mm]: BL = 3.60 (3.20–3.47, 3.33–3.78), ThL = 1.64 (1.42–1.64, 1.42–1.78), WL = 3.42 (3.02–3.33, 3.11–3.64), WW = 1.38 (1.20–1.38, 1.24–1.42).

*Indices*: arb = 4/3 (4/3), avd = 1.06 (0.83–1.11), adf = 2.57 (3.17–3.83), flw = 1.57 (1.57–2.00), FW/HW = 0.43 (0.38–0.45), ch/o = 0.12 (0.08–0.15), prorb = damaged (0.52–0.63), rcorb = damaged (0.25–0.29), vb = 1.20 (0.86–1.20), dcl = damaged (0.68–0.76), presctl = damaged (0.39–0.46), sctl = damaged (0.97–1.07), sterno = 1.23 (0.70–1.22), orbito = 0.56 (0.50–0.60), dcp = 0.56 (0.46–0.55), sctlp = 0.88 (0.88–1.00), C = 2.28 (1.94–2.16), 4c = 1.02 (0.98–1.24), 4v = 2.02 (1.85–2.32), 5x = 1.33 (1.29–1.73), ac = 2.30 (2.35–2.72), M = 0.53 (0.49–0.68), C3F = 0.91 (0.88–0.98).

##### Etymology.

A combination of the Greek words: “*melas*” (= black) + “*gaster*” (= abdomen), referring to the abdomen nearly black.

##### Distribution.

China (Yunnan).

#### Scaptodrosophila nigricostata sp. n.

Taxon classificationAnimaliaORDOFAMILIA

http://zoobank.org/BFBB670B-256F-4F32-AD2A-8E5423C8EF7A

Figs 5E–H , 13

##### Type material.

Holotype ♂ (SCAU, No. 128253): CHINA: Baihualing, Baoshan, Yunnan, alt. 1400 m, 7.vi.2011, ex tussocks, HW Chen. Paratypes: CHINA: 1♂, 4♀ (SCAU, Nos 128248–51, 128374), HW Chen, JJ Gao, same data as holotype; 1♂ (SCAU, No. 128254), Wangtianshu, Mengla, Yunnan, 21°47'N, 101°63'E, alt. 580 m, 23.iv.2007, HW Chen.

##### Diagnosis.

This species is similar to *S. nigripecta* sp. n. in the shape of the paramere and the pattern on the mesonotum (Fig. 13C, D), but can be distinguished from the latter by having the mesonotum mostly yellow (Fig. 5G), and the aedeagus slender and rod-like (Fig. 13C, D); see under that species.

##### Description.

Male and female: *Head* (Fig. 5E): frons yellowish brown with a brown band anteriorly. Pedicel yellowish brown; first flagellomere yellowish brown. Facial carina yellowish brown, short and broad, as 1/3 length as face.

*Thorax* (Fig. 5F, G): mesonotum with three dark brown longitudinal stripes medially and sublaterally. Acrostichal setulae in ca. 8–10 irregular rows. Scutellum yellowish brown, dark brown near basal scutellar setae, pale at tip. Pleura brown to dark brown.

*Abdomen* (Fig. 5H): all tergites yellowish brown with dark brown caudal bands, the caudal bands on tergites II and III narrowed medially.

*Male terminalia* (Fig. 13A–D): epandrium with ca. 15 setae near posterior and ventral margins per side. Surstylus with seven peg-like prensisetae. Hypandrium with a pair of paramedian setae and pubescence medially. Paramere with seven sensilla medially and pubescence distally. Aedeagus lacking pubescence.

*Female terminalia* (Fig. 13E): oviscapt with one subapical trichoid ovisensillum, 16, 11 and 5 peg-like ovisensilla per side on ventral, dorsal and apical margins, respectively.

*Measurements* [holotype (paratypes range in 2♂, 4♀), in mm]: BL = 3.38 (2.86–3.33, 2.86–3.29), ThL = 1.60 (1.47–1.64, 1.33–1.56), WL = 3.33 (3.20–3.29, 2.67–3.33), WW = 1.29 (1.20–1.24, 1.07–1.33).

*Indices*: arb = 4/3 (4/3), avd = 1.00 (0.88–1.43), adf = 2.67 (3.20–4.00), flw = 1.67 (1.83–3.00), FW/HW = 0.42 (0.41–0.47), ch/o = 0.13 (0.05–0.13), prorb = damaged (0.46–0.64), rcorb = damaged (0.27–0.36), vb = 1.00 (0.50–1.00), dcl = 0.77 (0.55–0.69), presctl = 0.44 (0.28–0.43), sctl = damaged (0.88–1.18), sterno = 0.73 (0.56–0.92), orbito = 0.56 (0.44–0.56), dcp = 0.47 (0.40–0.50), sctlp = 0.94 (0.86–1.00), C = 2.11 (2.20–2.59), 4c = 1.23 (0.90–1.18), 4v = 2.15 (2.11–2.29), 5x = 1.80 (1.67–2.20), ac = 2.37 (2.43–2.57), M = 0.68 (0.50–0.65), C3F = 0.96 (0.89–0.96).

##### Etymology.

A combination of the Latin words: “*niger*” + “*costa*”, referring to the black pleura.

##### Distribution.

China (Yunnan).

#### Scaptodrosophila nigripecta sp. n.

Taxon classificationAnimaliaORDOFAMILIA

http://zoobank.org/8C4FF708-2A9A-4B00-9021-5F0BF170A9A0

Figs 6A–D , 14

##### Type material.

Holotype ♂ (SCAU, No. 128264): CHINA: Wangtianshu, Mengla, Yunnan, 21°47'N, 101°63'E, alt. 760 m, 22.iv.2007, HW Chen. Paratypes: CHINA: 4♂ (SCAU, Nos 128265–68), Wangtianshu, Mengla, Yunnan, 21°47'N, 101°63'E, alt. 760 m, 22.iv.2007, 9.v.2012, HW Chen.

##### Diagnosis.

This species is similar to *S. protenipenis* sp. n. in the aedeagus with pubescence (Fig. 14D), but can be distinguished from the latter by having the paramere apically divided into two triangular lobes in lateral view (Fig. 14D); aedeagus with a cluster of pubescence on small apical part in lateral view (Fig. 14D); see under that species.

##### Description.

Male and female: *Head* (Fig. 6A): frons yellowish brown with a brown band anteriorly (Fig. 6A). Pedicel brown; first flagellomere yellowish. Facial carina brown.

*Thorax* (Fig. 6B, C): mesonotum brown, with two yellowish brown longitudinal stripes submedially. Acrostichal setulae in ca. 10–12 irregular rows. Scutellum brownish, dark brown near basal scutellar setae, pale at tip. Pleura dark brown.

*Abdomen* (Fig. 6D): all tergites brownish with dark brown caudal bands, the caudal bands on tergites II and III narrowed medially.

Male terminalia (Fig. 14A–D): epandrium with ca. 17 setae near posterior and ventral margins per side. Surstylus with 6–7 peg-like prensisetae. Hypandrium with a pair of paramedian setae and pubescence medially. Paramere with six sensilla medially and pubescence distally. Aedeagus slightly curved, with thinner pubescence ventrally.

*Measurements* [holotype (paratypes range in 4♂), in mm]: BL = 2.80 (2.58–2.76), ThL = 1.20 (1.20–1.24), WL = 2.49 (2.40–2.71), WW = 0.89 (0.93–1.02).

*Indices*: arb = 4/3 (4/3), avd = 0.94 (0.86–0.95), adf = 3.60 (3.40–3.80), flw = 1.60 (1.80–2.00), FW/HW = 0.43 (0.30–0.41), ch/o = 0.09 (0.07–0.12), prorb = 0.59 (0.57–0.71), rcorb = 0.27 (0.32–0.33), vb = 1.00 (1.00–1.17), dcl = damaged (0.62–0.70), presctl = damaged (0.31–0.40), sctl = damaged (0.96), sterno = damaged (1.00–1.05), orbito = 0.50 (0.50–0.63), dcp = 0.46 (0.39–0.50), sctlp = 0.92 (0.85–0.92), C = 1.77 (1.71–1.80), 4c = 1.26 (1.25–1.31), 4v = 2.17 (2.20–2.25), 5x = 2.00 (1.80–2.00), ac = 3.55 (2.71–3.75), M = 0.65 (0.64–0.70), C3F = 0.87 (0.80–0.92).

##### Etymology.

A combination of the Latin words: “*niger*” + “*pectus*”, referring to the black thorax.

##### Distribution.

China (Yunnan).

#### Scaptodrosophila obscurata sp. n.

Taxon classificationAnimaliaORDOFAMILIA

http://zoobank.org/068FE33E-3079-4857-8860-78DFC48EE2CE

Figs 6E–H , 15

##### Type material.

Holotype ♂ (SCAU, No. 128347): CHINA: Wangtianshu, Mengla, Yunnan, 21°47'N, 101°63'E, alt. 760 m, 9.v.2012, HW Chen. Paratypes: CHINA: 1♂ (SCAU, No. 128348), same data as holotype; 20♂, 15♀ (SCAU, Nos 128349–368), Menglun, Mengla, Yunnan, 21°55'N, 101°16'E, alt. 570 m, 3–4.xi.2001, HW Chen; 1♂ (SCAU, No. 128369), Hesong, Menghai, Yunnan, alt. 1940 m, 6.v.2012, HW Chen.

##### Diagnosis.

This species differs from the other known species of this group in having the paramere with a hook-shaped projection basoventrally (Fig. 15D), and the aedeagus apically acute in lateral view (Fig. 15D).

##### Description.

Male and female: *Head* (Fig. 6E): frons brown and glossy. Pedicel brown; first flagellomere yellowish. Facial carina brown and glossy.

*Thorax* (Fig. 6F, G): mesonotum brown, with two yellowish brown longitudinal stripes submedially. Acrostichal setulae in ca. 10–12 irregular rows. Scutellum brown, dark brown near basal scutellar setae, pale at tip. Pleura dark brown.

*Abdomen* (Fig. 6H): tergites II to V yellow anteromedially, with black caudal bands; tergite VI yellowish brown.

*Male terminalia* (Fig. 15A–D): epandrium with ca. 16 setae near posterior and ventral margins per side. Surstylus with 5–6 peg-like prensisetae. Hypandrium with a pair of paramedian setae and pubescence medially. Paramere with four sensilla medially. Aedeagus lacking pubescence.

*Female terminalia* (Fig. 15E): oviscapt with one subapical trichoid ovisensillum, 15, 11 and 5 peg-like ovisensilla per side on ventral, dorsal and apical margins, respectively.

*Measurements* [holotype (paratypes range in 6♂, 1♀), in mm]: BL = 3.47 (2.93–3.42, 3.42), ThL = 1.60 (1.38–1.60, 1.64), WL = 2.98 (2.71–2.98, 3.11), WW = 1.16 (1.07–1.20, 1.24).

*Indices*: arb = 4/3 (4/3), avd = 0.95 (0.94–1.05), adf = 3.50 (3.29–4.00), flw = 1.67 (1.67–2.00), FW/HW = 0.38 (0.38–0.43), ch/o = 0.09 (0.05–0.12), prorb = 0.65 (0.32–0.63), rcorb = 0.23 (0.19–0.38), vb = 1.00 (0.83–1.00), dcl = damaged (0.58–0.73), presctl = damaged (0.23–0.42), sctl =0.88 (0.92–1.06), sterno = 0.65 (0.59–0.85), orbito = 0.50 (0.46–0.67), dcp = 0.43 (0.40–0.46), sctlp = 0.93 (0.88–1.07), C = 2.20 (1.98–2.30), 4c = 1.03 (1.00–1.21), 4v = 1.98 (1.88–2.26), 5x = 1.77 (1.69–2.00), ac = 2.16 (2.10–2.73), M = 0.58 (0.60–0.69), C3F = 0.83 (0.84–0.93).

##### Etymology.

From the Latin word “*obscurata*” (= dark), referring to the thorax dark.

##### Distribution.

China (Yunnan).

#### Scaptodrosophila protenipenis sp. n.

Taxon classificationAnimaliaORDOFAMILIA

http://zoobank.org/9A15E25D-846D-41D4-A5A7-DDE46D59A132

Figs 7A–D , 16

##### Type material.

Holotype ♂ (SCAU, No. 128269): CHINA: Hesong, Menghai, Yunnan, alt. 1940 m, 6.v.2012, HW Chen. Paratypes: CHINA: 6♀ (SCAU, Nos 128270–75), HW Chen, JJ Gao, same data as holotype; 12♂, 19♀ (SCAU, Nos 128277–95, 128375), Hesong, Menghai, Yunnan, alt. 1940 m, 16.iv.2010, K Liu, JM Lu, ZF Shao, SJ Yan; 1♂ (SCAU, No. 128276), Baihualing, Baoshan, Yunnan, alt. 1400 m, 7.vi.2011, HW Chen, JJ Gao.

##### Diagnosis.

Paramere apically divided into two round lobes in lateral view (Fig. 16D); aedeagus with dense pubescence in lateral view (Fig. 16D).

##### Description.

Male and female: *Head* (Fig. 7A): frons brownish with a brown band anteriorly. Pedicel brown; first flagellomere yellowish. Facial carina yellowish brown, short, as 1/3 length as face.

*Thorax* (Fig. 7B, C): mesonotum yellowish brown, with four brown longitudinal stripes. Acrostichal setulae in ca. 10–12 irregular rows. Scutellum yellowish brown, dark brown near basal scutellar setae, pale at tip. Pleura dark brown.

*Abdomen* (Fig. 7D): tergites II to V brownish with dark brown caudal bands, the caudal bands on tergite II narrowed dorsomedially; tergite VI brownish.

*Male terminalia* (Fig. 16A–D): epandrium with ca. 17 setae near posterior and ventral margins per side. Surstylus with six peg-like prensisetae. Hypandrium with a pair of paramedian setae and pubescence medially. Paramere with seven sensilla medially and pubescence distally. Aedeagus with pubescence ventrally.

*Female terminalia* (Fig. 16E): oviscapt with one subapical trichoid ovisensillum, 16, 11 and 5 peg-like ovisensilla per side on ventral, dorsal and apical margins, respectively.

*Measurements* [holotype (paratypes range in 5♂, 4♀), in mm]: BL = 3.33 (3.16–3.51, 3.33–3.38), ThL = 1.60 (1.56–1.69, 1.60–1.73), WL =3.42 (3.16–3.33, 3.33–3.51), WW = 1.38 (1.29–1.38, 1.33–1.47).

*Indices*: arb = 4/3 (4/3), avd = 1.00 (0.90–1.00), adf = 3.17 (2.17–3.80), flw = 1.83 (1.43–2.20), FW/HW = 0.41 (0.40–0.44), ch/o = 0.10 (0.09–0.13), prorb = 0.62 (0.50–0.63), rcorb = 0.31 (0.27–0.40), vb = 1.00 (0.67–1.20), dcl = damaged (0.67–0.72), presctl = 0.51 (0.37–0.47), sctl = damaged (0.98–1.15), sterno = 0.73 (0.66–0.79), orbito = 0.56 (0.50–0.60), dcp = 0.46 (0.46–0.53), sctlp = 0.94 (0.81–1.00), C = 1.98 (1.86–2.33), 4c = 1.11 (1.02–1.23), 4v = 2.04 (2.00–2.28), 5x = 1.44 (1.32–1.67), ac = 2.78 (2.21–2.79), M = 0.51 (0.52–0.63), C3F = 0.92 (0.88–0.96).

##### Etymology.

A combination of the Latin words: “*protensus*” + “*penis*”, referring to the protruded aedeagus.

##### Distribution.

China (Yunnan).

#### Scaptodrosophila rhina sp. n.

Taxon classificationAnimaliaORDOFAMILIA

http://zoobank.org/0AAD1B38-B17E-404C-B5F1-B1BDC732F377

Figs 7E–H , 17

##### Type material.

Holotype ♂ (SCAU, No. 128255): CHINA: Menglun, Mengla, Yunnan, 21°55'N, 101°16'E, alt. 570 m, 3, 4.xi.2001, HW Chen. Paratypes: CHINA: 7♀ (SCAU, Nos 128256–61, 128376), same data as holotype; 1♂ (SCAU, No. 128263), Baihualing, Baoshan, Yunnan, alt. 1400 m, 7.vi.2011, HW Chen.

##### Diagnosis.

This species differs from the other species of the *brunnea* group in the mesonotum being yellowish brown, lacking a longitudinal stripe (Fig. 7F), the pleura being yellowish brown (Fig. 7G), and the paramere distally divided in lateral view (Fig. 17D).

##### Description.

Male and female: *Head* (Fig. 7E): frons yellowish brown with a brown band anteriorly. Pedicel brown; first flagellomere yellowish. Facial carina yellowish brown.

*Thorax* (Fig. 7F, G): acrostichal setulae in ca. 10–12 irregular rows. Scutellum yellowish brown, dark brown around basal scutellar setae, pale at tip. pleura yellow, with brown patches.

*Abdomen* (Fig. 7H): tergites II to V yellow, with dark brown caudal bands on tergites III to V, the caudal bands on tergite III and IV interrupted medially; tergite VI dark brown to black.

*Male terminalia* (Fig. 17A–D): epandrium with ca. 16 setae near posterior and ventral margins per side. Surstylus with five peg-like prensisetae. Hypandrium with a pair of paramedian setae, lacking pubescence. Paramere with five sensilla and pubescence medially. Aedeagus with pubescence ventrally.

*Female terminalia* (Fig. 17E): oviscapt with three subapical trichoid ovisensilla, 16, 15 and 5 peg-like ovisensilla per side on ventral, dorsal and apical margins, respectively.

*Measurements* [holotype (paratypes range in 1♂, 1♀), in mm]: BL = 3.33 (3.42, 3.33), ThL = 1.51 (1.60, 1.60), WL = 3.20 (3.20, 3.24), WW = 1.29 (1.20, 1.29).

*Indices*: arb = 4/3 (4/3), avd = 0.83 (0.80–0.95), adf = 3.00 (3.00–3.80), flw = 1.67 (1.67–1.83), FW/HW = 0.43 (0.42–0.43), ch/o = 0.11 (0.11) , prorb = damaged (damaged), rcorb = 0.31 (damaged), vb = 1.00 (1.00–1.17), dcl = 0.75 (0.64), presctl = 0.40 (0.43), sctl = damaged (damaged), sterno = 0.71 (0.73), orbito = 0.56 (0.44–0.56), dcp = 0.45 (0.47), sctlp = 0.93 (0.88), C = 2.14 (2.10–2.15), 4c = 1.07 (1.02–1.10), 4v = 2.12 (2.07–2.24), 5x = 1.67 (1.79–1.80), ac = 2.44 (2.39–2.56), M = 0.61 (0.60–0.64), C3F = 0.96 (0.91–0.96).

##### Etymology.

From the Greek words: “*rhnios*”, referring to the facial carina large and prominent.

##### Distribution.

China (Yunnan).

### Key to examined species of the *brunnea* group

**Table:** 

1	Body brown; carina large; arista exceptionally large, with 4–5 curved long dorsal branches and 3 ventral branches in addition to terminal bifurcation (Figs 3–7A, E)	***brunnea* group**...**2**
2	Hypandrium lacking pubescence medially (Figs 12, 17C)	**3**
–	Hypandrium pubescent medially (Figs 8–11C, 13–16C)	**4**
3	Mesonotum yellowish brown, with four brown longitudinal stripes sublaterally (Fig. 5B); pleura dark brown (Fig. 7G); paramere expanded and not divided distally in lateral view (Fig. 12D)	***S. melanogaster* sp. n.**
–	Mesonotum yellowish brown, lacking longitudinal stripe (Fig. 7F); pleura yellowish brown (Fig. 7G); paramere distally divided in lateral view (Fig. 17D)	***S. rhina* sp. n.**
4	Aedeagus pubescent ventrally (Figs 13D, 14D, 16D)	**5**
–	Aedeagus lacking pubescence (Figs 8–11D, 15D)	**7**
5	Mesonotum brown, with two yellowish brown longitudinal stripes submedially (Fig. 6B); paramere apically divided into two triangular lobes in lateral view (Fig. 14D)	***S. nigripecta* sp. n.**
–	Mesonotum yellowish brown, with three or four longitudinal stripes (Figs 5F, 7B); paramere apically do not divided into two triangular lobes in lateral view	**6**
6	Mesonotum yellowish brown, with four brown longitudinal stripes (Fig. 7B); aedeagus with dense pubescence in lateral view (Fig. 16D)	***S. protenipenis* sp. n.**
–	Mesonotum yellowish brown, with three dark brown longitudinal stripes medially and sublaterally (Fig. 5F); aedeagus slender rod-like (Fig. 13C, D)	***S. nigricostata* sp. n.**
7	Mesonotum yellowish brown, with a brownish longitudinal stripe (Fig. 3B, F)	**8**
–	Mesonotum brown, with two or three brownish longitudinal stripes (Figs 4B, F, 6F)	**9**
8	Mesonotum with a longitudinal stripe on 1/3 posterior (Fig. 3F); pleura dark brown (Fig. 3G); paramere with a small projection basally (Fig. 9D)	***S. pressobrunnea* (Tsacas & Chassagnard)**
–	Mesonotum yellowish brown, with a brown longitudinal stripe medially (Fig. 3B); paramere lacking projection subbasally (Fig. 8D)	***S. parabrunnea* (Tsacas & Chassagnard)**
9	Mesonotum brown, with three yellowish brown longitudinal stripes (Fig. 4B); pleura brownish (Fig. 4C); paramere pubescent distally (Fig. 10D)	***S. scutellimargo* (Duda)**
–	Mesonotum brown, with two yellowish brown longitudinal stripes (Figs 4F, 6F); pleura dark brown (Figs 4G, 6G); paramere lacking pubescence distally (Figs 11C, D, 15C, D)	**10**
10	Frons brown and glossy (Fig. 6E); scutellum brown (Fig. 6F); paramere with hook-shaped projection basoventrally (Fig. 15D)	***S. obscurata* sp. n.**
–	Frons brownish and dull (Fig. 4E); scutellum brownish (Fig. 4F); paramere lacking projection basoventrally (Fig. 11D)	***S. maculata* sp. n.**

## Discussion

The specimens identified as *S. pressobrunnea* and *S. scutellimargo* putatively in this study mostly match the original descriptions, especially in the male terminalia described and illustrated by Tsacas and Chassagnard (1976) and Duda (1924), respectively, while differences are found in the color patterns on mesonotum (lacking longitudinal stripes) in Tsacas and Chassagnard (1976) and Duda (1924). Actually, color patterns of mesonotum can varied intraspecifically in the family Drosophilidae. Similar cases had been reported in *Leucophenga piscifoliacea* Huang & Chen, 2013 and *L. rectifoliacea* Huang & Chen, 2013. Thus, specimens of *S. pressobrunnea* and *S. scutellimargo* putative in this study were recognized as the known species.

The integration of morphological and DNA-based approaches has revealed an effective way to improve accuracy for species identification (Dayrat 2005; Lumleyand Sperling 2010; Padial and De La Riva 2010). In the present study, we try to use the molecular data to text the putative, morpho-species. Each of the new species *S. melanogaster* sp. n., *S. nigricostata* sp. n., *S. nigripecta* sp. n., *S. obscurata* sp. n., *S. protenipenis* sp. n. and *S. rhina* sp. n. is supported as monophyletic in the NJ tree, and their maximum intraspecific distances are lower than the minimum interspecific distances. In addition, “simple pure characters” are all successfully found in these putative species. Thus, the validity of these seven new species described in the present study was confirmed by the DNA data and morphological research.

It is noteworthy that no “simple pure character” is found for *S. maculata* sp. n. in the character-based analyses, and the smallest interspecific distance in the *brunnea* group is detected between *S. maculata* sp. n. and *S. parabrunnea* (3.3%), which is above the 3% (or 2%) sequence divergence threshold (Hebert et al. 2003a, b, 2004). Actually, the “simple pure character” are not a perfect fix in some case, especially for species in the *brunnea* group with extremely similar *COI* haplotypes, or in cases hybridization and introgression will influence the success of mitochondrial identification methods, which had been observed in turtles of the genus *Graptemys* (Reid et al. 2011), as species often lacked identifying characters simply because of the lack of available variation in *COI*. Although *S. maculata* sp. n. is morphologically similar to *S. parabrunnea*, they can be distinguished easily by the shape of facial carina (Figs 3A, 4E), paramere and aedeagus (Figs 8D, 11D). In the phylogenetic analyses, the NJ tree recovered them as distinct clades (Fig. 1). Therefore, *S. maculata* sp. n. putative was designated as new species.

Dayrat (2005) has previously proposed the use of different sources of evidence in taxonomic practice (i.e. geography, ecology, reproductive isolation, phylogeography, comparative morphology, population genetics, development, behavior), which is now called ‘integrative taxonomy’. In fact, wide overlap between intra- and interspecific distances (0–15.5%) has been repeatedly observed in Diptera (Meier et al. 2006), indicating the necessity of using additional marker(s), and incorporating other sources of information (e.g., geographical and ecological) in species discrimination in this order (Huang et al. 2013).

## Supplementary Material

XML Treatment for Scaptodrosophila parabrunnea

XML Treatment for Scaptodrosophila pressobrunnea

XML Treatment for Scaptodrosophila scutellimargo

XML Treatment for Scaptodrosophila maculata

XML Treatment for Scaptodrosophila melanogaster

XML Treatment for Scaptodrosophila nigricostata

XML Treatment for Scaptodrosophila nigripecta

XML Treatment for Scaptodrosophila obscurata

XML Treatment for Scaptodrosophila protenipenis

XML Treatment for Scaptodrosophila rhina
